# LncRNA HClnc1 facilitates hepatocellular carcinoma progression by regulating PKM2 signaling and indicates poor survival outcome after hepatectomy

**DOI:** 10.1002/cam4.6117

**Published:** 2023-05-22

**Authors:** Qian Zhu, Zhengqing Lei, Chang Xu, Zheng Zhang, Zeqian Yu, Zhangjun Cheng, Pengfeng Xiao, Shufeng Li, Weiping Yu, Jiahua Zhou

**Affiliations:** ^1^ Department of Hepatobiliary Surgery Zhongda Hospital, Southeast University, School of Medicine Nanjing Jiangsu China; ^2^ Department of Hepatobiliary and Pancreatic Surgery Zhongnan Hospital of Wuhan University Wuhan Hubei China; ^3^ First Department of Biliary Surgery, Eastern Hepatobiliary Surgery Hospital Second Military Medical University Shanghai China; ^4^ State Key Laboratory of Bioelectronics, National Demonstration Center for Experimental Biomedical Engineering Education, School of Biological Science and Medical Engineering Southeast University Nanjing Jiangsu China; ^5^ Department of Biochemistry and Molecular Biology Medical School of Southeast University Nanjing Jiangsu China; ^6^ Department of pathophysiology Southeast University School of Medicine Nanjing Jiangsu China

**Keywords:** HClnc1, hepatocellular carcinoma, long noncoding RNA, PKM2, prognosis, Warburg effect

## Abstract

**Aim:**

Long noncoding RNAs (lncRNAs) are key mediators with a wide range of pathophysiological functions, but their role in human hepatocellular carcinoma (HCC) is still unclear.

**Methods:**

An unbiased microarray study evaluated a novel lncRNA, HClnc1, that is linked to the development of HCC. In vitro cell proliferation assays and an in vivo xenotransplanted HCC tumor model were performed to determine its functions, followed by antisense oligo‐coupled mass spectrometry to identify HClnc1‐interacting proteins. To study relevant signaling pathways, in vitro experiments were performed, including chromatin isolation by RNA purification, RNA immunoprecipitation, luciferase, and RNA pull‐down assay.

**Results:**

HClnc1 levels were considerably greater in patients with advanced tumor‐node‐metastatic stages, and it was found to be inversely connected to survival rates. Moreover, the proliferative and invasive potential of the HCC cells was attenuated by HClnc1 RNA knockdown in vitro, while HCC tumor growth and metastasis were found to be reduced in vivo. HClnc1 interacted with pyruvate kinase M2 (PKM2) to prevent its degradation and thus facilitated aerobic glycolysis and PKM2‐STAT3 signaling.

**Conclusions:**

HClnc1 is involved in a novel epigenetic mechanism of HCC tumorigenesis and PKM2 regulation. HClnc1 is not only a more accurate prognostic indicator of HCC but also a potential therapeutic target for HCC treatment.

## 1 INTRODUCTION

Because of difficulties in diagnosis and poor prognosis, hepatocellular carcinoma (HCC) is considered to be the most common cause of cancer‐associated mortalities across the globe.^1^ Approximately 60%–70% of HCC patients are ineligible for curative resection due to late diagnosis and inaccurate prognosis. New and more precise molecular biomarkers are therefore urgently needed. Long noncoding RNAs (lncRNAs) are emerging RNA species that considerably contribute to carcinogenesis and metastasis.^2^, ^3^, ^4^, ^5^ In HCC, the lncRNAs IDH1‐AS1, HULC, DANCR, UFC1, ATB, and PVT1 are upregulated to promote carcinogenesis through several mechanisms, such as epigenetic modification, miRNA sponging, and transcriptional regulation.^3^, ^6^, ^7^, ^8^, ^9^, ^10^ UCA1 promotes HCC cell proliferation and malignant transformation of hepatocyte‐like stem cells.^11^

Although the roles of lncRNAs in tumorigenesis have been established, their mechanisms of action in cancer‐related processes, such as the Warburg effect, remain poorly understood. Considered a hallmark of cancer, the Warburg effect is essential for providing energy and biosynthetic building blocks to sustain the high proliferation rates of cancer cells.^12^, ^13^ The Warburg effect is implemented by switching the pyruvate kinase M1 isoform to M2 to shift pyruvate from oxidation to lactic acid fermentation. Pyruvate kinase M2 (PKM2) may act as a protein kinase in the nucleus, regulating several genes and cascades related to malignancies, particularly HIF‐1, Oct‐4, cyclin D1, and beta‐catenin. Aside from its critical involvement in metabolic reprogramming, PKM2 may act as a multifunctional signaling molecule that facilitates cancer progression.^10^, ^12^ PKM2, a regulator of STAT3 signaling in colorectal cancer, was recognized as a vital downstream target of HClnc1 via experimental screening and validation. Although PKM2 activity is crucial for cancer cell fate determination, the underlying lncRNA processes remain little explored.

Herein, we found HClnc1, a new lncRNA that is frequently expressed in HCC tissues and is linked with poor survival outcomes in HCC patients. By regulating the PKM2 protein, HClnc1 enhances HCC cell proliferation and tumor development. In conclusion, HClnc1 acts as a more reliable prognostic marker and a promising therapeutic target in HCC treatment.

## 2 MATERIALS AND METHODS

### 2.1 Cell lines and tumor samples

The HCC cell lines including HCCLM3, LO2, Hep3B, SMMC7721, Huh7, and HepG2 were obtained from the Chinese Academy of Sciences Cell Bank. These cell lines were grown in DMEM enriched with 10% FBS at 37°C with a continuous supply of 5% CO_2_.

Five HCC and paired noncancerous tissue samples were collected for lncRNA microarray analysis. Quantitative real‐time PCR (qRT‐PCR) and survival analyses were utilized to validate the microarray results in 60 (Cohort 1) and 120 (Cohort 3) pairs of HCC and surrounding tissue samples, accordingly. In situ hybridization (ISH) with a particular digoxin‐labeled HClnc1 probe on tissue arrays containing 80 pairs (Cohort 2) of HCC and adjacent tissue samples was used to assess the expression level of HClnc1 in tissues. A total of 238 HCC tissues (Cohort 4) were analyzed to determine the expression of both HClnc1 and PKM2. In addition, the clinicopathologic characteristics were compared to demonstrate the balance of baseline between the four cohorts. Appendix 1 contains the complete experimental designs. Between March 1 and August 31, 2008, all samples were taken from patients undergoing hepatectomy at the Eastern Hepatobiliary Surgery Hospital. The Ethics Committee approved the use of human tissue, and all patients gave informed consent in written form to take part in the study. Post excision, tumor tissues and para‐cancerous healthy tissues (at least 2 cm distant from the tumor edge) were obtained from the same patient. Then the underlined tissues were snap‐frozen and preserved in liquid nitrogen until use. Table S1 lists the clinical features of the HCC patients. The Edmondson grading system^14^ was used for tumor grade classification. The seventh edition of the International Union Against Cancer's tumor‐node‐metastasis (TNM) classification system was used to determine tumor stage. Micrometastasis were characterized as tumors that could be recognized microscopically and were located near the primary tumor border.

### 2.2 qRT‐PCR

Detailed instructions and sequences of probes/primers are provided in Appendix 1and Table S2, respectively.

### 2.3 Cell proliferation, cell cycle, apoptosis, and migration

The Cell Counting Kit‐8 (CCK‐8) assay was conducted to evaluate cellular proliferation. In a 96‐well plate, control and treated HCC cells were grown at an initial density of 3.0 × 10^4^ cells per well. Following the manufacturer's instructions, the reaction product was measured post 24 h of culturing. Flow cytometry was used to examine the cell cycle and apoptotic process of the cell. Appendix 1 contains step‐by‐step instructions.

Transwell experiments were carried out in 24‐well Transwells (Millipore, 8‐μm pore size) that had been pretreated with Matrigel (BD Biosciences). The transfection of HClnc1 siRNA or control siRNA was carried out into logarithmic phase cells, followed by harvesting and seeding the cells (at a density of 1 × 10^5^ cells/mL) into the chamber in serum‐free media. Each well received medium enriched with 20% FBS. The cells on the inner surface of the chamber were removed after 24‐h incubation, while the cells that had migrated to the outer surface of the membrane were fixed, stained, and counted.

### 2.4 Assays for drug sensitivity to 5‐fluorouracil and oxaliplatin

The CCK‐8 assay was used to examine cellular proliferation. The transfection of control and siRNA‐transfected cells was carried out in 24‐well plates at an initial density of 3 × 10^4^ cells/well before being treated with 5‐fluorouracil and oxaliplatin dosages. At various time intervals, a CCK‐8 reagent (10 μL/well) was introduced into the cells. Post 2 h, the culture media was removed, and then cells fixation was performed. By measuring the absorbance at 450 nm, the reaction product was determined.

### 2.5 In situ hybridization (ISH)

ISH was used to determine the expression level of HClnc1 in tissues using a digoxin‐labeled HClnc1 probe on tissue arrays comprising 80 pairs of HCC and surrounding tissue samples. A quantitative scanning approach was used to identify the staining and expression of HClnc1 with an Aperio ImageScope V12 from Leica,^15^, ^16^ and the positivity value 100× represented the expression of HClnc1. The sequence of the HClnc1 probe for ISH was digoxin‐5′‐TGCACTCTGTTATCTGGAACT‐3′‐digoxin.

### 2.6 RNA fluorescent in situ by hybridization (FISH)

Using the RiboTM Fluorescent In Situ Hybridization Kit, a FISH assay was performed (RiboBio Company). Exiqon Company developed and synthesized the HClnc1 and U6 probes, which were tagged with Cy3 fluorescent dye. A confocal laser scanning microscope (LSM 780, Zeiss) was used to measure fluorescence.

### 2.7 Immunohistochemistry (IHC)

IHC was performed as described previously.^17^ At 4°C, the incubation of tissue sections was carried out with EZH2 or a PKM2 antibody for 24 h. Immunostaining was performed using the ChemMate DAKO EnVision Detection Kit (Dako).

### 2.8 Pyruvate kinase activity and lactate detection

A Pyruvate Kinase (PK) Assay Kit (Abcam) and a Lactate Assay Kit (Abcam) were used to evaluate PKM2 activity and lactate formation in HCC cells, accordingly.

### 2.9 Luciferase reporter assay

STAT3 transcriptional activity was measured using pGL4.47[Luc2P/SIE/Hygro] (Promega), which contains five copies of the SIS‐inducible element (SIE) that promotes transcription of the luciferase reporter gene luc2P. The plasmids were transiently transfected into HEK‐293Ta cells as indicated, with the pRL‐TK Renilla luciferase plasmid serving as a control to account for variances between wells. The Dual‐Luciferase Reporter Assay System (Promega) was used to conduct a luciferase reporter assay 48 h post‐transfection.

### 2.10 In vitro transcription and translation

The HClnc1 cDNA was cloned into pBluescript II SK (+) downstream of the T7 promoter using a directed cloning method and linearized with Kpn I. In vitro transcription of linear pBluescript II SK (+)‐HClnc1 (468 ng) was performed (Epicentre), followed by in vitro translation of 700 ng of pure HClnc1 RNA using biotinylated leucine tRNA (Epicentre). The BrightStar BioDetect Kit was used to detect biotinylated proteins (Ambion). As positive and negative controls, LacZ mRNA and mock‐translated samples (without an RNA template) were utilized, accordingly.

### 2.11 RNA pull‐down assay

The CytoSelect cell transformation assay (Cell Biolab, Inc.) was used to perform RNA pull‐down assays, which were carried out according to the manufacturer's instructions. For RNA pull down, the samples were treated with biotinylated RNA and streptavidin beads. Details are available in Appendix 1.

### 2.12 LC–MS analysis, database search, and protein evaluation

We compared HClnc1 with antisense HClnc1 pull‐down eluates to identify specific HClnc1 interactors. The bands of the HClnc1 pull‐down sample were chosen, excised, and subjected to LC–MS analysis. Proteins identified by at least two peptides were included in the analysis. A detailed description is provided in Appendix 1.

### 2.13 RNA immunoprecipitation (RIP)

A biotin‐labeled HClnc1 probe (5′‐TGCACTCTGTTATCTGGAACT‐3′‐biotin) was synthesized by Exiqon Company, and Magna RIPTM RNA‐Binding Protein Immunoprecipitation Kit (Millipore) was employed for RNA immunoprecipitation studies.^18^

### 2.14 Chromatin isolation by RNA purification (ChIRP)

An online probe designer (www.singlemoleculefish.com) was used to examine the ChIRP probes HClnc1 antisense DNA (asDNA) and HClnc1 sense DNA (sDNA). With an 18‐carbon spacer arm, oligonucleotides were biotinylated at the 3′ end. Huh7 cells were collected and exposed to ChIRP as described previously.^19^

### 2.15 Silencing and overexpression of HClnc1

GenePharma generated siRNA to target HClnc1. Huh7 and HCCLM3 cells were transfected with the HClnc1 siRNA or HClnc1‐overexpressing plasmid as described previously.^20^ The sequence of the HClnc1 siRNA1 &siRNA2 were 5′‐GCAAGGAUUUAGGGUUCUATT‐3′ and 5′‐CCUGUCAAAUGCAGGCCAUTT‐3′, respectively.

### 2.16 Preparation of HCC‐bearing nude mouse model and experiments in vivo

Four‐week‐old male BALB/c nude mice were acquired from Nanjing Institutes for Biological Sciences' Experimental Animal Centre and bred as previously described.^21^, ^22^ Two weeks after subcutaneous incubation of different numbers of HCC cancer cells, an adenovirus encoding HClnc1 shRNA1, HClnc1 shRNA2, or a control shRNA or PBS was intratumorally injected every 3 days for 2 weeks. The tumors' size and weight were assessed, and then the expression of Ki67 was evaluated via IHC. Following that, the tumors' growth and invasion were observed. The approval for the experimental methods was provided by the Southeast University School of Medicine's Institutional Animal Care and Use Committee. Appendix 1 contains the details of the in vivo investigations.

### 2.17 Generation of tissue microarray

As previously described,^23^ a tissue microarray (TMA) was generated. Hematoxylin and eosin stains were used to examine all of the samples histologically. Away from necrotic and hemorrhagic tissue, representative sections on paraffin blocks were marked. Each tumor had two 1.0‐mm cores excised, matched with non‐tumor tissue, and fixed on a fresh recipient block using a semiautomated array device (TMArrayer).

### 2.18 Generation of plasmid and adenovirus

Shanghai Obio Technology Company designed and generated the shRNAs (control shRNA, HClnc1 shRNA1/2, and PKM2 shRNA), as well as the HClnc1‐overexpressing adenovirus and all of the plasmids.

### 2.19 Statistical analysis

A detailed description is provided in Appendix 1.

## 3 RESULTS

### 3.1 LncRNAs profiling in HCC tumor tissues

**FIGURE 1:**
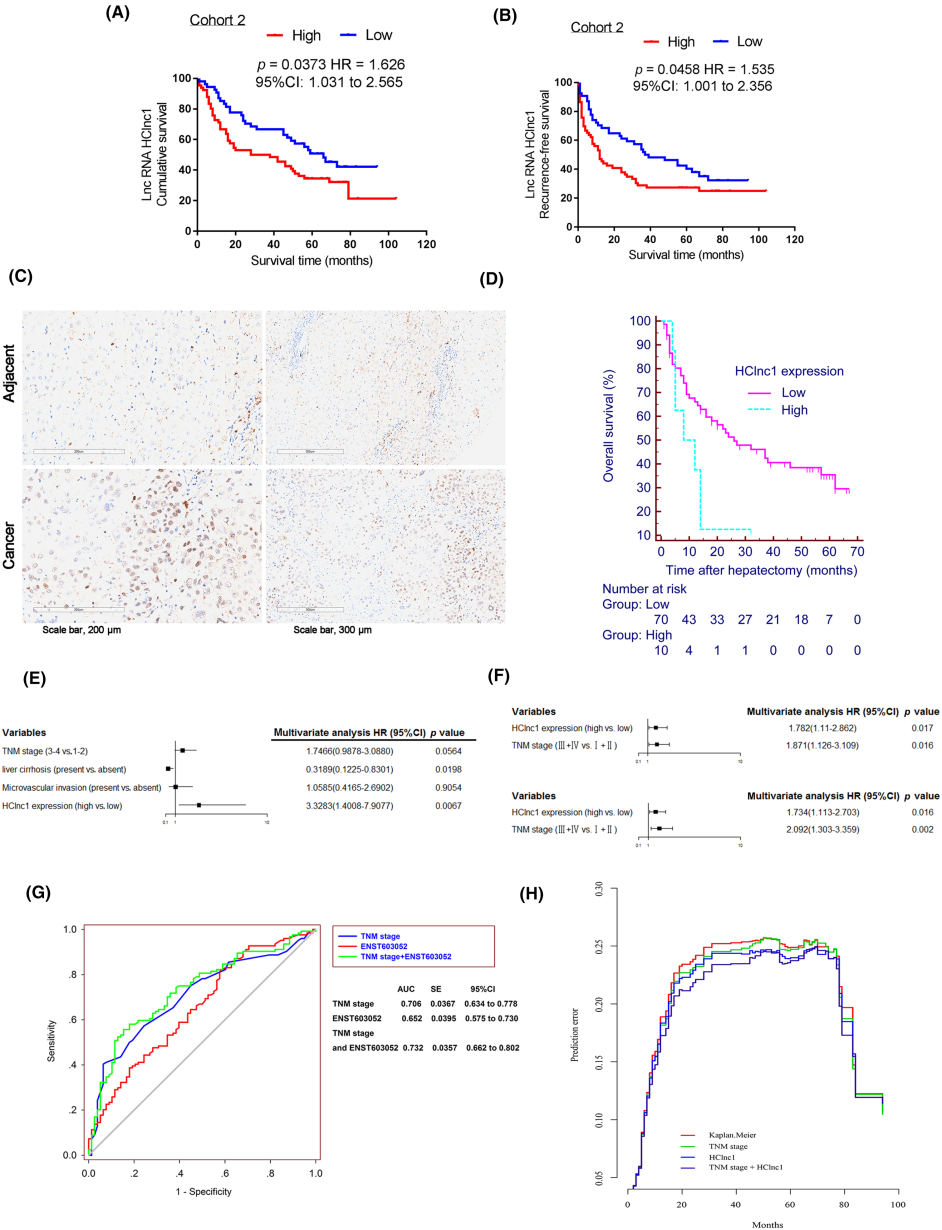
LncRNA candidate ENST00000603052 (HClnc1) is clinically related to hepatocellular carcinoma (HCC). (A) and (B) represent the analyses and comparison of the survival rates among patients associated with lower and higher expression levels of HClnc1 among 120 patients with HCC (Cohort 3); log‐rank test. (C) ISH was used to analyze representative images of HClnc1 expression in HCC and in Cohort 2; *n* = 80. (D) Survival analysis was performed to compare the levels of HClnc1 expression in tumor patients in Cohort 2; log‐rank test. (E) A multivariate analysis was performed in Cohort 2. (F) A multivariate analysis was used in Cohort 3. (G) ROC analysis of HClnc1 was performed in Cohort 3. (H) Prediction error curves for various predictors in Cohort 3. For the 5‐year follow‐up, the apparent error (AE) and 10‐fold cross‐validated cumulative prediction error (PE) were calculated.

To profile the expression of lncRNA in HCC tissues, we evaluated five pairs of HCC tissues with a lncRNA microarray (GSE112613, Figure S1A). The 10 most upregulated lncRNAs in HCC tissues were identified, and their expression changes were determined in a cohort of 60 HCC patients (Cohort 1) (Figure S1B). Among the identified lncRNAs, six (ENST00000603052, NR_125715, BC005081, NR_024206, NR_024205, ENST00000558391) were verified in a larger independent cohort of 120 HCC patients (Cohort 3). To examine whether these lncRNAs also correlate with patient survival, we performed a Kaplan–Meier analysis and identified a lncRNA, ENST00000603052, that correlated with reduced overall survival (OS) and recurrence‐free survival (RFS) in HCC patients (Figure 1A,B; Figure S1C). We named this new lncRNA hepatocellular carcinoma‐associated lncRNA1 (HClnc1). Further analysis with a *K*‐adaptive partitioning statistical (kaps) algorithm^24^, ^25^ identified an HClnc1 expression cutoff value of 8.369 that distinguished the OS of these HCC patients with the highest significance (Figure 1A). The increased expression of HClnc1 in HCC tissues was further verified by independent HCC datasets (GEO numbers GSE45436, GSE55092, GSE62232; extracted in Figure S1D).

We further verified and analyzed HClnc1 expression by ISH in additional 80 paraffin‐embedded liver tumors and surrounding tissues (Cohort 2). In terms of the association between HClnc1 expression and the clinicopathologic characteristics and prognosis of HCC patients, we obtained results similar to those of Cohort 2, it was worth pointing out that univariate analyze demonstrated that liver cirrhosis was considerably linked with OS and RFS (Figure 1C‐E, Table S3, Figure S1E–G). Collectively, these data identified a novel lncRNA highly correlated with HCC progression and mortality.

### 3.2 Link between HClnc1 expression and the clinicopathological characteristics and poor survival of HCC

A clinicopathological association analysis of Cohort 3 indicated that 66 patients with high HClnc1 expression (values above the average, ΔCt = 8.847, range: 1.703–0.210) had significantly larger tumor sizes (*p* = 0.015) and higher AFP levels (*p* < 0.001; Table 1) than 54 patients with low HClnc1 expression (values below the average, ΔCt: 4.528, range: 1.417–0.193). However, no correlation was observed between HClnc1 expression and gender, tumor number, serum albumin, bilirubin, HBe antigen, HBs antigen, ALT, or AFT, the presence of encapsulation, metastasis, TNM stage, micro‐ or macrovascular invasion, or Edmondson grade (Table 1).

**TABLE 1:** Clinical features of 120 HCC patients based on HClnc1 expression.

Variables	All	HClnc1 subgroup^a^		*p* value
		Low	High	
All cases	120	54	66	
Age (years)	50.6 ± 11.3	54.1 ± 11.0	47.8 ± 10.8	0.002*
Age: >55/<55	42/78	27/27	15/51	0.003
Gender: male/female	106/14	50/4	56/10	0.304**
HBs antigen: +/−	102/18	49/5	53/13	0.182
Anti‐HCV: +/−	3/117	1/53	2/64	0.197
Liver cirrhosis: +/−	71/49	37/17	34/32	0.089
Albumin (g/L)	41.1 ± 4.44	40.8 ± 4.57	41.2 ± 4.35	0.618
Albumin (g/L): >40/≤40	65/55	31/23	34/32	0.645
Bilirubin (μmol/L)	15.5(5.2–48.3)	14.5(5.2–48.3)	15.9(8.0–38.6)	0.325
Bilirubin (μmol/L): >17/≤17	32/88	13/41	19/47	0.709
HBe antigen: +/−	30/90	17/37	13/53	0.204
Tumor size (cm)	6.0(1.0–22.0)	5.0(1.0–22.0)	7.0(1.2–20.0)	0.015*
Tumor size (cm): >5/≤5	66/54	26/28	40/26	0.238
No. of tumors: solitary/multiple	96/24	45/9	51/15	0.551
Edmondson grade				
Well	7	3	4	0.162**
Moderately	3	3	0	
Poorly	108	48	60	
Undifferentiated	2	0	2	
Micro‐vascular invasion: +/−	69/51	31/23	38/28	1.000
Macro‐vascular invasion: +/−	14/106	6/48	8/58	1.000
Satellite: +/−	17/103	8/46	9/57	1.000
Encapsulation: complete/−	72/48	34/20	38/28	0.680
TNM stage				
I	42	22	20	0.206**
II	47	17	30	
III	29	15	14	
IV	2	0	2	
ALT (U/L)	52.3(9.6–411)	54.9(11.9–411)	50.3(9.6–376)	0.437
ALT (U/L): >40/≤40	72/48	36/18	36/30	0.246
AFP (μg/L)	390(2.5–63,159)	14.2(2.5–1000)	1000(113–63,159)	<0.001*
AFP (μg/L): >20/≤20	87/33	21/33	66/0	<0.001**

*Note*: Normally distributed data are shown as a ratio or mean ± SD; non‐normally distributed data are shown as median (IQR).

^a^ The threshold was set at the median expression level. Values below the 50th percentile indicate reduced expression of lncRNA HClnc1 (in 54 patients). Values at or above the 50th percentile represent elevated lncRNA HClnc1 expression (in 66 patients).

* *p* < 0.05 by *t*‐test.

** *p* < 0.05 by Fisher exact test.

To determine the prognostic value of HClnc1 on OS and RFS in HCC patients, we conducted univariate and multivariate Cox regression analyses by adjusting for possible prognostic factors. First, univariate analysis was conducted to explore the potential prognostic variables, which demonstrated that tumor size, macrovascular invasion, number of tumors, TNM stage, AFP, and HClnc1 expression were considerably linked with OS and RFS (Tables S4 and S5). As tumor size, macrovascular invasion, and the number of tumors were included in the TNM stage, only TNM stage, AFP, and HClnc1 expression were assessed using a multivariate Cox proportional hazards model, which indicated that HClnc1 expression was an independent risk factor for the OS and RFS of HCC patients post hepatectomy (Figure 1F, Tables S6 and S7, *p* = 0.017 for OS, *p* = 0.016 for RFS, respectively). Moreover, to analyze potential interactions between HClnc1 and the TNM stage, a postestimation Wald test was performed to obtain a *p*‐value. We found that the interaction effect of HClnc1 and TNM stage on OS and RFS was significant (Tables S8 and S9, for OS, *p* = 0.0342; for RFS, *p* = 0.0041, respectively). The results indicated that the prediction effect of HClnc1 on the OS and RFS of HCC might be different across the 7th TNM subclassification. As shown in Figure S1H and I, HClnc1 was also significantly associated with RFS (*p* = 0.0078) and OS (*p* = 0.0013) in 39 HCC patients at TNM Stages III&IV. Furthermore, ROC evaluation indicated that the AUC of HClnc1 and the seventh TNM‐based model combined (0.732) was elevated than that of the TNM‐based model alone (0.706, *p* = 0.03, Figure 1G). Based on the Brier score, the predicted probability of being wrong was lower when using a combination of HClnc1 and TNM stage than the TNM stage alone in Cohort 3 (Figure 1H). The combining of HClnc1 and the TNM stage is more reliable than the TNM stage alone in predicting OS, according to these findings.

### 3.3 HClnc1 is an authentic LncRNA

To investigate whether HClnc1 has protein‐ or peptide‐coding potential, we examined its protein‐coding potential by two powerful online tools, Coding‐Non‐Coding Index (CNCI)^26^ and PhyloCSF.^27^ HClnc1 had a negative protein‐coding potential score (−1.1556) and a very low codon substitution frequency score (−877.7), suggesting that it is not likely to encode peptide products (Figure S1J–L). HClnc1 also did not contain any valid Kozak sequences (score: −0.012).^26^ The prediction results were further verified by ribosome profiling,^28^ which revealed no/minimal ribosome binding of HClnc1 (Figure S1M), and also by an in vitro translation assay, which showed a lack of protein/peptide products (Figure S1N). These data collectively indicate that HClnc1 is a lncRNA without protein‐coding potential.

### 3.4 HClnc1 regulates HCC proliferation, invasion, and resistance to chemotherapy drugs

**FIGURE 2:**
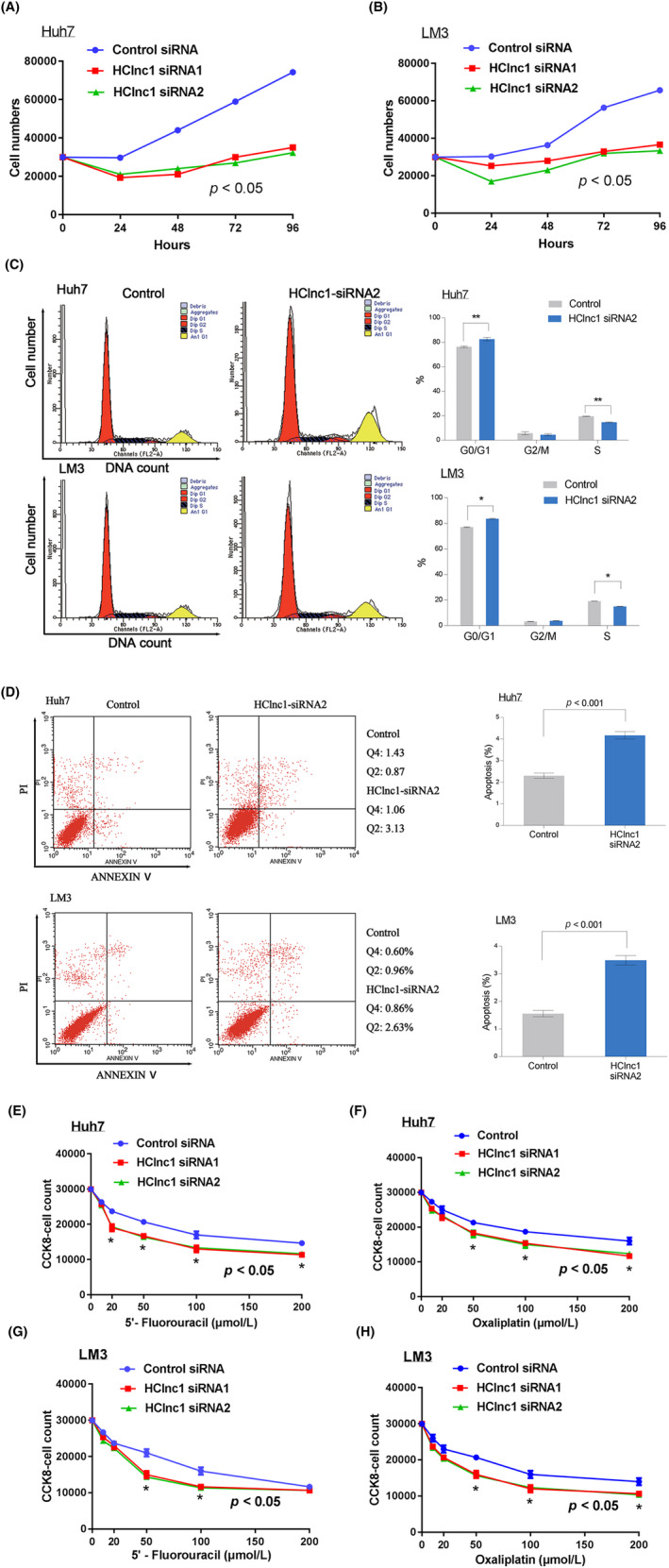
Silencing of HClnc1 inhibited proliferation and induced apoptosis and cell cycle arrest in HCC cells. (A) and (B) represent the transfection of Huh7 and HCCLM3 cells, accordingly. Both types of cells were transfected with HClnc1 siRNA1/2. Each experiment was performed in triplicate (*n* = 3). Nonparametric Mann–Whitney test. (C) Flow cytometry was used to assess the cell cycle distribution of Huh7 and HCCLM3 cells transfected for 24 h and stained with PI. Representative and quantitative results are shown. *n* = 3; * *p* < 0.05. (D) Apoptosis of Huh7 and HCCLM3 HCC cells 3 days after transfection with HClnc1 siRNA was assessed by flow cytometry. (E–H) Dose–response curve of a representative experiment showing relative 5′‐fluorouracil and oxaliplatin sensitivity determined by CCK8. Huh7 and LM3 cells were transfected with control or HClnc1 siRNA1/2 and were treated with 5′‐fluorouracil and oxaliplatin; *n* = 3, nonparametric Mann–Whitney test, compared with control siRNA group. Illustrative scatter plots and quantitative data are presented. *n* = 3; **p < 0.01.

**FIGURE 3:**
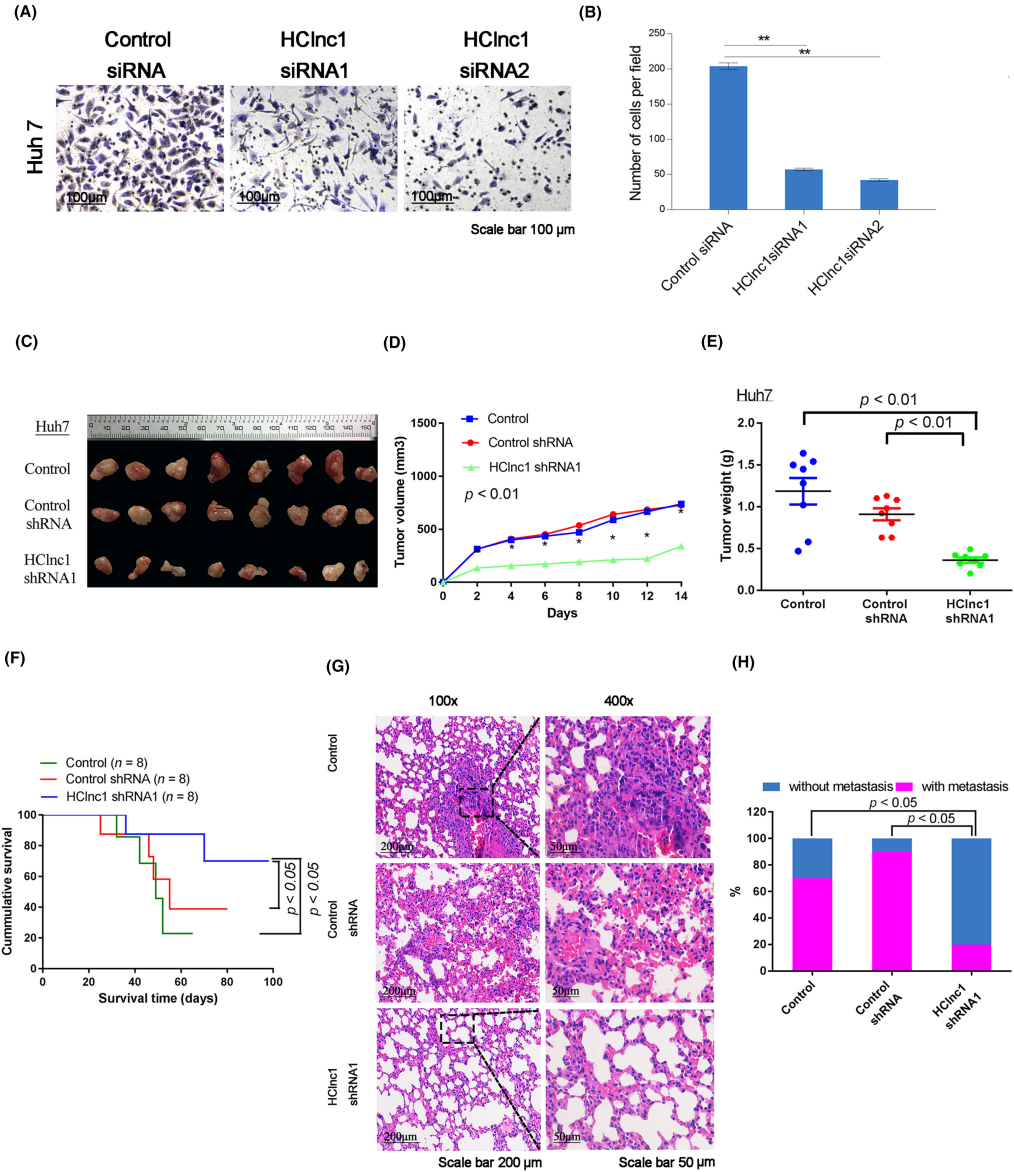
HClnc1 is an oncogenic lncRNA in HCC. (A) Tumors in nude mice exhibiting PBS transfected HCC cells, control shRNA adenovirus, or HClnc1 shRNA1 adenovirus (*n* = 8); (B) evaluating tumor volume following HClnc1 shRNA1 adenovirus treatments in the xenograft mouse model; *n* = 8; *p < 0.05, nonparametric Mann–Whitney test. In (C), tumor weight in mice was assessed after various treatments; *n* = 8, the nonparametric Mann–Whitney test was used. (D, E) A Transwell Matrigel invasion experiment was done in Huh7 cells that had been treated with siRNA for HClnc1 or a control siRNA (*n* = 3). (F) Mice exhibiting HCC cells transfected with PBS, HClnc1, shRNA1 adenovirus, or control shRNA adenovirus, survival analysis was done; *n* = 10, log‐rank test. (G) and (H) represent hematoxylin and eosin staining (100× or 400× magnification) and summarize the obtained results on tumor lung foci in nude mice 14 weeks post HClnc1 shRNA1 adenovirus injection; *n* = 10. **p < 0.01.

Consistent with the increased expression of HClnc1 in advanced HCC patients (Figure 1G), HClnc1 expression was elevated in the advanced HCC cell lines HCCLM3 and Huh7 relative to the less aggressive cell lines SMMC7721 and Hep3B (Figure S2A–D), suggesting separate mechanisms of HClnc1. Indeed, HClnc1 knockdown significantly suppressed Huh7 and HCCLM3 cell proliferation, leading to cell arrest at the G2/M checkpoint and susceptibility to apoptosis (Figure 2A–D, Figure S2E,F). Consistent with the reduced proliferation and impaired drug resistance (Figure 2E–H), the invasion capacity of HCC cells was also significantly suppressed by HClnc1 knockdown (Figure 3A,B; Figure S2U). These data collectively indicated that HClnc1 contributed to the proliferative and invasive potential of the HCC cells. Consistent with these in vitro effects, xenotransplantation of HClnc1‐knockdown Huh7 and HCCLM3 cells dramatically reduced tumor growth and weight, as well as lung and liver metastasis (Figure 3C–E; Figure S2G), leading to increased OS time (Figure 3F ,H). Ki‐67 staining demonstrated that tumor proliferation was largely reduced (Figure S2H,I). These data indicate that HClnc1 is required for HCC tumorigenesis and progression.

To determine whether HClnc1 is sufficient to drive HCC cell proliferation and invasion, the less aggressive HCC cells SMMC7721 and Hep3B with lower HClnc1 expression were infected with lentivirus containing HClnc1 cDNA (Figure S2D and S2J–K). HClnc1 increased the proliferative and invasive potential of SMMC7721 as well as Hep3B cells (Figure S2L–U). Moreover, fewer metastatic foci were found in the lungs and livers in nude mice with HClnc1 shRNA adenovirus, leading to a longer OS time (Figure S2V,W). Consistent with the in vitro effects, the tumor growth rate, weight, and the number of foci of pulmonary metastasis were significantly increased in mice transplanted with HClncl‐overexpressing SMMC7721 cells (Figure S2O). These data indicate that HClnc1 is sufficient to promote the proliferative and metastatic capability of HCC cells in vitro and in vivo.

### 3.5 The LncRNA HClnc1 regulates PKM2 stability

**FIGURE 4:**
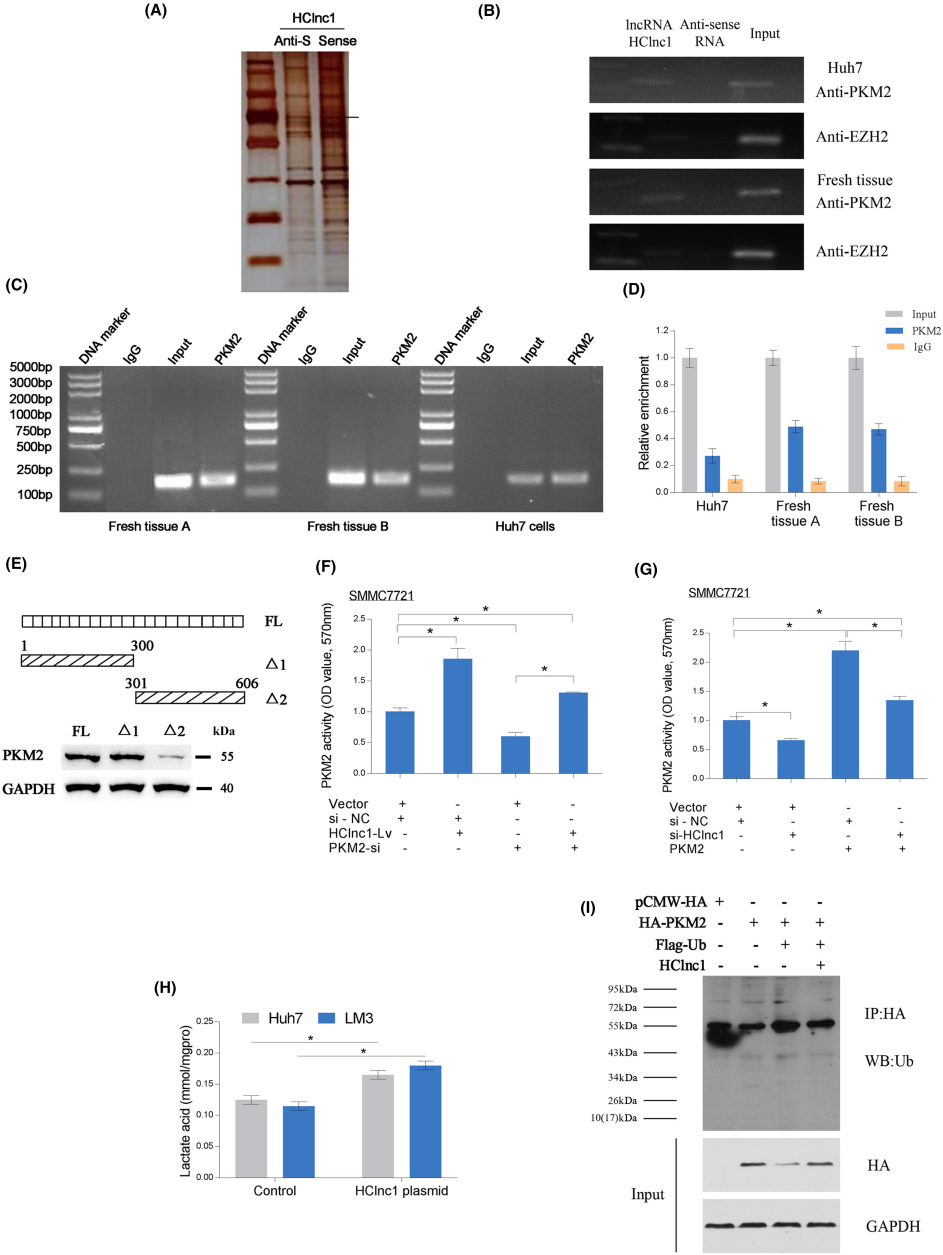
HClnc1 promotes HCC progression by interacting with PKM2. SDS‐PAGE gels of proteins bound to anti‐sense RNA and HClnc1 (A and B). The PKM2 protein was determined using mass spectrometric evaluation of the band shown by the arrow. Two separate RNA pull‐down experiments from Huh7 cellular extracts or HCC tissue were analyzed by immunoblotting. An antibody against the EZH2 protein was used as a negative control. (C) and (D) show the results of RIP studies evaluated on Huh7 cell extracts or two fresh HCC tissues using an antibody targeting PKM2. The SYBR‐Green technique was employed to evaluate qRT‐PCR experiments on the isolated RNA, and the enrichment of lncRNA HClnc1 was evaluated by adjusting the input. (E) Immunoblotting of PKM2 in samples pulled down by full‐length (FL) or truncated HClnc1 (Δ1: 1–300, Δ2: 301–606); *n* = 3. (F, G) HClnc1 enhanced pyruvate kinase activity by regulating PKM2 in SMMC7721 cells. (H) A lactic acid detection kit (JianCheng) was employed to detect the accumulation of lactic acid in HCCLM3 and Huh7 cells transfected with LV‐NC or LV‐lncRNA‐HClnc1, according to the manufacturer's instructions. A two‐sample *t*‐test was used to determine the significance of the differences. (I) Ubiquitous alteration of PKM2 was prevented by ectopic HClnc1 expression. HClnc1, HA‐PKM2, and Flag‐Ub plasmids were transfected into SMMC7721 cells, which were then exposed to MG132 (20 mmol/L) for 3 h. Following immunoprecipitation of HA‐PKM2 with an anti‐HA antibody, ubiquitinated PKM2 was checked out by immunoblotting with an anti‐Ub antibody. *p < 0.05; **p < 0.01.

To screen potential protein targets of HClnc1, HClnc1‐interacting proteins in Huh7 cells were concentrated by biotinylated antisense HClnc1 oligo and identified by mass spectrometry. Among all five identified candidates, only PKM2 was verified to interact with HClnc1 (Figure 4A–D, Table S10). To identify the PKM2‐interacting region of HClnc1, we constructed and biotinylated two fragments of HClnc1(1–300Δ1, 301–606Δ2) and used them in the pull‐down assay with Huh7 cell lysates. We found that the 5′ fragment of HClnc1 mediated the interaction with PKM2. HClnc1 is directly bound to PKM2 at its 5′ end (1–300 bp) (Figure 4E). Further experimental results showed that PKM2 kinase activity and the level of lactate were considerably increased by ectopic HClnc1 expression, whereas HClnc1 knockdown attenuated pyruvate kinase activity and lactate formation in HCC cells (Figure 4F–H). To confirm these data, we evaluated the effect of HClnc1 on PKM2 ubiquitination in HCC cells. While HClnc1 overexpression did affect PKM2 mRNA levels (Figure S3), ectopic HClnc1 expression could increase the stability of PKM2 via reducing its ubiquitination. Together, these results demonstrate that HClnc1 increases the levels of PKM2 by binding and increasing its stability. (Figure 4I).

### 3.6 HClnc1 activates the PKM2/STAT3 pathway

**FIGURE 5:**
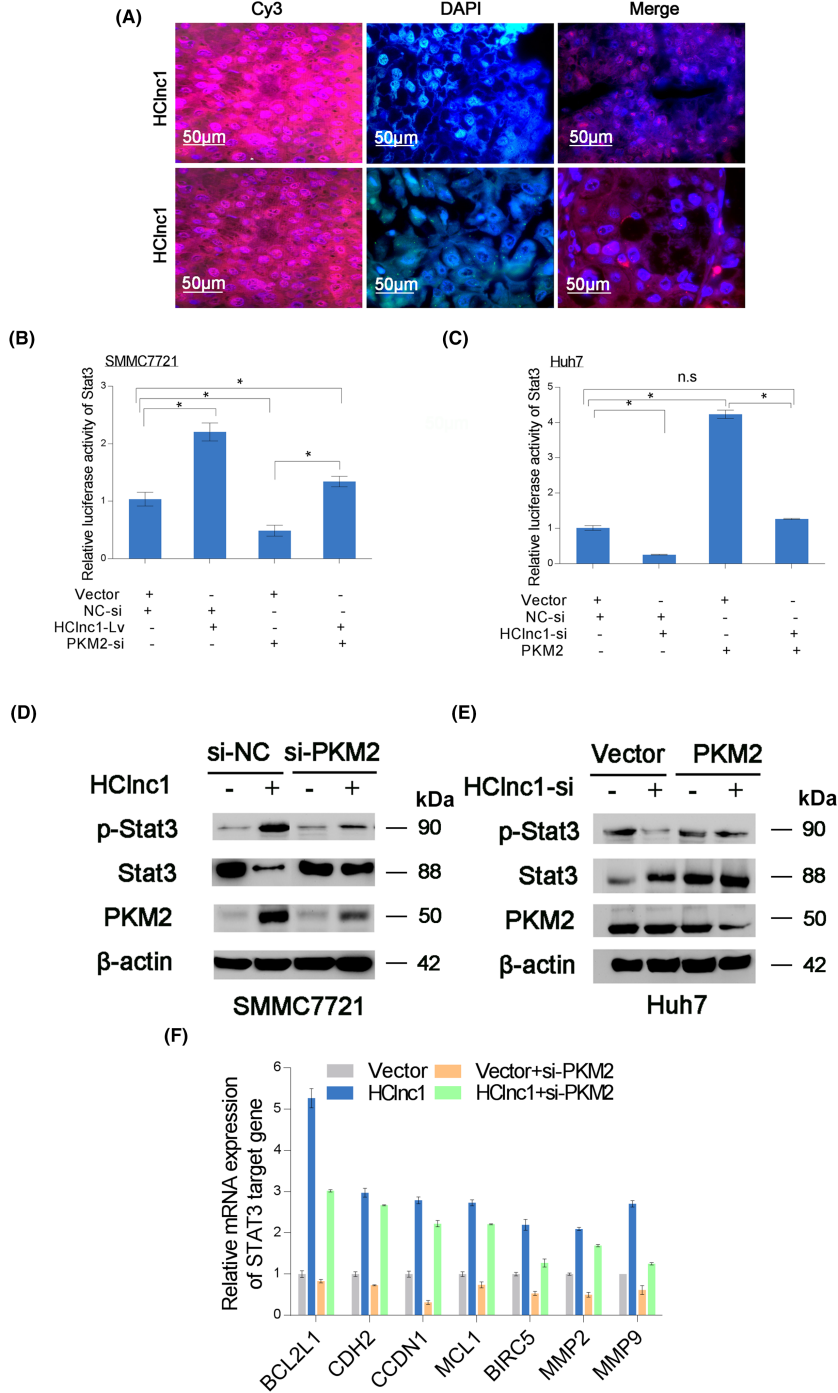
HClnc1 promotes HCC progression by activating STAT3 signaling and enhancing glycolysis through PKM2. (A) RNA FISH (*n* = 160) demonstrated the subcellular localization of HClnc1 and U6 in HCC and neighboring healthy tissue. Original magnification, 630×. Scale bars, 20 μm. (B, C) HClnc1 triggered the STAT3 cascade through PKM2. The impacts of HClnc1 and PKM2 on the STAT3 cascade were evaluated using a luciferase assay. (D, E) HClnc1 promoted STAT3 phosphorylation (Tyr705) via PKM2 in HCC cells. (F) HClnc1 stimulated the expression of STAT3 cascade downstream targets via PKM2. qRT‐PCR was used to determine the mRNA levels of these genes. *p < 0.05, n.s., not significant.

Gene set enrichment analysis indicated that the STAT3 pathway correlates with HClnc1 (Figure S4), suggesting that nuclear PKM2, a STAT3 modulator during tumorigenesis,^29^ is involved in the mechanisms of action of HClnc1. Indeed, nuclear PKM2 was significantly upregulated in HClnc1‐overexpressing HCC tissues (Figure 5A). A reporter assay indicated that STAT3 reporter activity was dramatically increased in HClnc1‐overexpressing HCC cells. Similarly, HClnc1 knockdown inhibited STAT3 reporter activity (Figure 5B,C). High expression of HClnc1 elevated endogenous STAT3 phosphorylation at Tyr705 residue, which was prevented by PKM2 knockdown, these results showed consistency with the reporter experiments (Figure 5D,E). Similarly, knocking down HClnc1 hindered STAT3 phosphorylation, which was restored by overexpressing PKM2 (Figure 5D,E). STAT3 downstream targets (CDH2, BCL2L1, MCL1, CCND1, MMP2, BIRC5, and MMP9) were likewise elevated in HClnc1‐overexpressing HCC cells, and their expression was considerably lowered by PKM2 knockdown, which was consistent with the underlined findings, as depicted in Figure 5F. These findings show that HClnc1 triggers the STAT3 cascade through the PKM2.

### 3.7 HClnc1 combined with PKM2 predicts HCC progression

**FIGURE 6:**
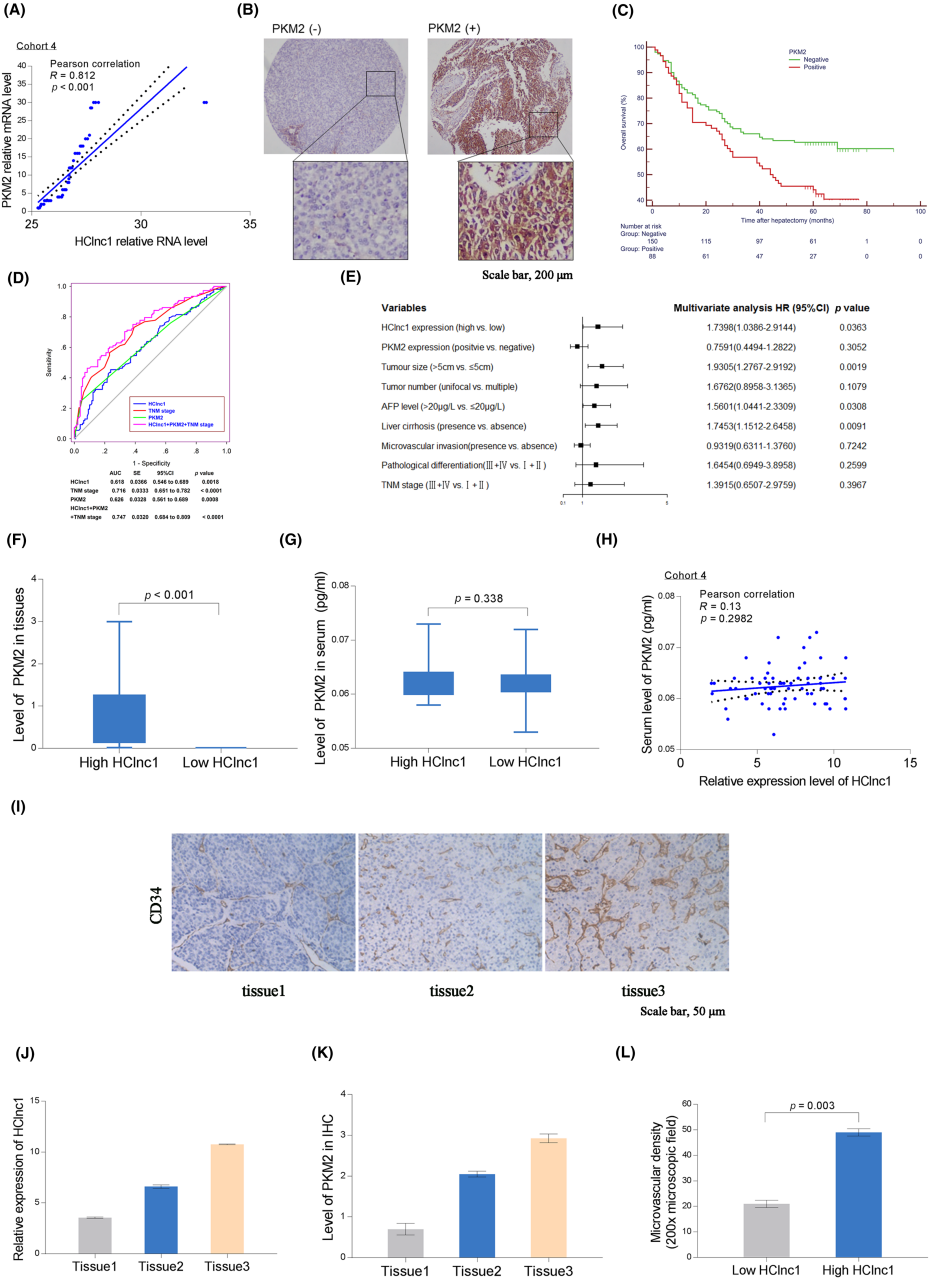
HClnc1 in combination with PKM2 predicts HCC progression. (A) Correlation between *PKM2* and HClnc1 RNA levels in 238 human HCC tissues. (B) Representative IHC staining for PKM2 in HCC tissues; *n* = 238. (C) The association between PKM2 and OS is shown in 238 patients with HCC; log‐rank test. (D) ROC analysis of the combined index of HClnc1, PKM2 and TNM stage in Cohort 4. (E) Multivariate analysis was performed on Cohort 4. (F, G) The level of PKM2 in tissues (not in serum) was considerably elevated in (G) in HCC patients with elevated HClnc1 expression relative to the control patients. (H) and (I) show the relative expression of HClnc1 that was plotted against the serum/tissue level of PKM2. (J–L) Representative images of the IHC evaluations are displayed, and the expression of HClnc1 and the serum level of PKM2 are presented in the bottom panel. Tissues were obtained from three patients with low, moderate, and increased HClnc1 expression and labeled as tissue 1, 2, and 3, accordingly.

Consistent with the HClnc1 profile in HCC patients, PKM2 was significantly overexpressed in 63.3% (150/238) of HCC patients (Cohort 4) and highly correlated with HClnc1 levels (Figure 6A,B). Furthermore, the OS and RFS rates of the PKM2^low^ patients were considerably higher than those of PKM2^high^ patients (both *p* < 0.05, Figure 6C; Figure S5A). A prognostic analysis using 238 HCC tissues (Cohort 4) confirmed that both HClnc1 and PKM2 expression levels were inversely correlated with OS and RFS (Figure S5A–C). Furthermore, ROC analysis indicated that the AUC of the combination of HClnc1‐based prediction, the PKM2‐based model, and the TNM‐based model (0.747) was elevated than that of the TNM‐based model alone (0.716, Figure 6D). The expression level of HClnc1 was found to be an independent predictor of HCC severity with substantial hazards ratios (HRs) in univariate and multivariate Cohort 4. It had a comparable predictive value to tumor size and AFP level (Figure 6E; Figure S5D). We further measured the microvascular density (MVD) and serum levels of PKM2 in 60 HCC tissues and corresponding serum samples from Cohort 1 by using IHC and ELISA, accordingly. We also analyzed the correlation between the HClnc1 expression level, angiogenesis, and the serum level of PKM2. It is worth pointing out that patients with high HClnc1 levels in serum samples did not show differential regulation of serum PKM2. However, a significant difference was found between the levels of HClnc1 and PKM2 in HCC tissues (r = 0.812, *p* < 0.001, Figure 6F–I), validating the link between PKM2 and HClnc1. Moreover, the serum level of PKM2 (*r* = 0.13, *p* = 0.2982, Figure 6H) was not directly linked with HClnc1 expression. PKM2 is released by tumor cells and facilitates angiogenesis,^30^ which is necessary for tumor growth and progression. Our results are all consistent with previous investigations showing that PKM2 is universally expressed in tumor cells and can play a role in regulating glycolysis and therefore the metabolic phenotype of cancer cells (Figure S5E). Additionally, the expression of HClnc1 was positively linked with MVD in tissues (*p* = 0.0136) (Figure 6I–L). Thus, upregulated HClnc1(35/60) was associated with high MVD and a high PKM2 level in primary HCC tissues. These results provide new evidence for the hypothesis that HClnc1 can enhance tumor‐inducing angiogenesis by elevating PKM2 expression.

**FIGURE 7:**
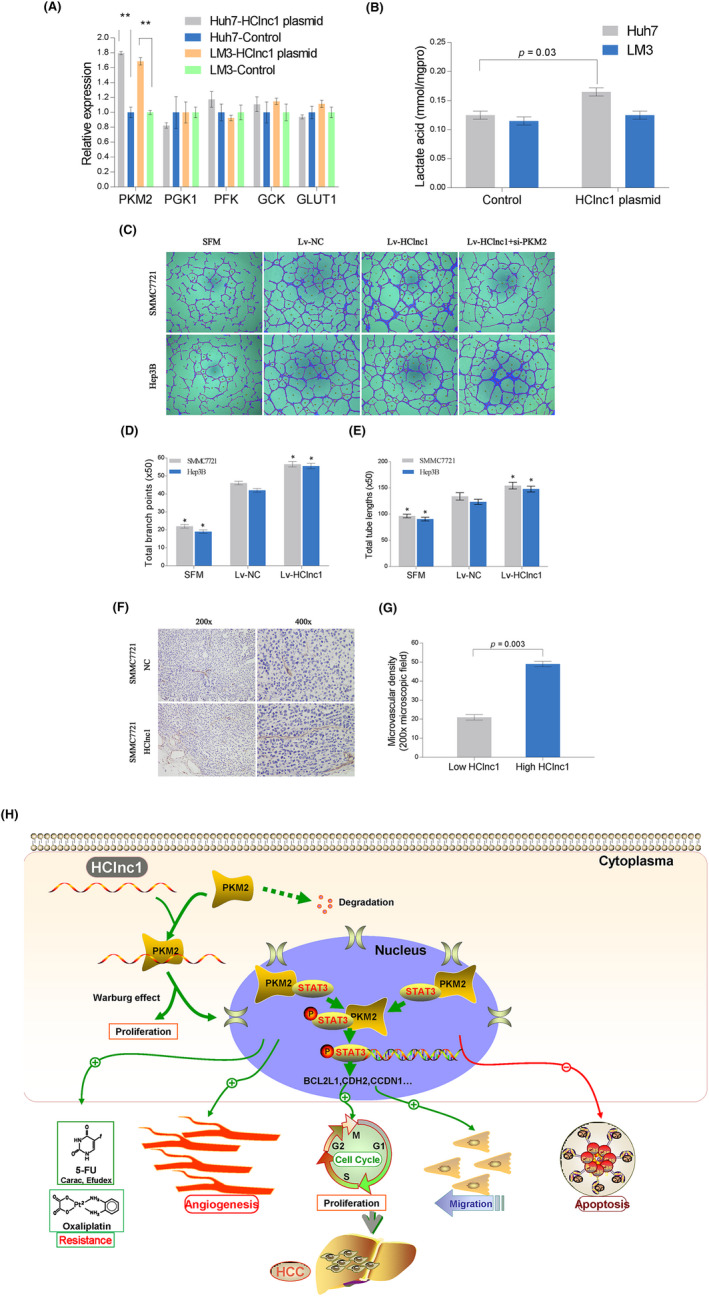
(A) Expression of key genes in glycolysis was detected using qRT‐PCR. Huh7 and HCCLM3 cells transfected with Lv‐lncRNA‐HClnc1 showed significant alteration of glycolytic markers compared with control cells. (B) The production of lactate acid in Huh7 and HCCLM3 cells transfected with LV‐lncRNA‐HClnc1 or LV‐NC was measured by a lactate acid detection kit (JianCheng) according to the manufacturer's instructions. The significance of differences was analyzed by the two‐sample *t*‐test. (C–E) Up‐regulated HClnc1 could activate HCC cells to promote the tube formation of HUVECs. Representative photographs of tube formation and the total branch points and total tube lengths are presented. SFM represents serum‐free medium. (F, G) Representative photographs of an IHC analysis of the tumors from the subcutaneous models are presented, and the micro‐vessel density (MVD) of the tissues was calculated. Data are shown as mean ± SD. The statistical differences were analyzed by Two‐sample *t*‐test. All groups were compared to the Lv‐NC control group for statistical comparisons. *p* < 0.05. H. Schematic representation of the biological mechanism by which HClnc1 modulates STAT3 signaling cascade and the Warburg effect (aerobic glycolysis) by elevating PKM2 activity in HCC cells.

We wondered whether the association between HClnc1 and PKM2 would play a vital role in this process. To test this hypothesis, we discovered significantly ectopic expression of the PKM2 gene was detected (Figure 7A). Then, we tested the levels of lactate acid described above. Our findings indicated that lactate acid was significantly increased in response (Figure 7B). Collectively, these results indicated that HClnc1 strengthened the function of PKM2 by inducing the expression of PKM2 in glycolysis. A tube formation assay was performed to further analyze the significance of this finding, with the TCM transfected with the LV‐ HClnc1, the human umbilical vein endothelial cells (HUVECs) developed more capillary‐like structures than did the negative control cells (Figure 7C–E), which was prevented by PKM2 knockdown. Therefore, we speculated that active angiogenesis facilitated by HClnc1 might account for the increased tumor growth and metastasis in vivo. Our results showed that the SMMC7721 cells overexpressing HClnc1 developed tumors with a higher MVD than did negative control cells (Figure 7F,G).

## 4 DISCUSSION

Numerous protein‐coding genes have been applied as prognostic biomarkers in the diagnosis and prognosis of HCC progression.^31^, ^32^ Although certain combinations of proteins showed improved precision, the general efficiency is still curbed by the fact that most protein‐coding gene‐related mechanisms are highly conserved and fundamentally altered during HCC progression.^33^ In contrast, lncRNAs, with much less conservation in sequence and function, provide more versatile regulatory mechanisms and valuable opportunities to establish more accurate diagnostic and prognostic combinations. In the current study, we demonstrated that HClnc1 in combination with the PKM2 protein was a more accurate predictor of HCC than conventional TNM. Our data established a new view of precision diagnosis by combining protein‐coding genes and long non‐coding genes. More importantly, whether HClnc1 can also be detected in plasma or circulating exosomes similar to PKM2, clarifying the correlation between circulating HClnc1 and HCC would provide a more convenient and dynamic parameter to monitor HCC progression.

In the current study, the kaps algorithm was employed to obtain an effective cutoff value for HClnc1 (8.369) to distinguish the outcomes of HCC patients. Although the exact optimal cutoff value should be verified in a broader group of patients, the above cutoff value is generally useful for the prognostic prediction of HCC patients. Moreover, we found that HClnc1 overexpression predicted the OS of advanced‐stage HCC patients. Further validation should be performed by a prolonged follow‐up of these HCC patients. Nevertheless, our data identified HClnc1 as a potential biomarker for risk prognostication and precision therapy screening of HCC patients after hepatectomy.

Several factors, such as MYC, Sp1, Sp3, miR‐130, miR‐326, and HIF‐1, regulate the expression of PKM2 either transcriptionally or post‐transcriptionally. Post‐translational processes, such as phosphorylation, sumoylation, acetylation, and prolyl hydroxylation all affect PKM2 function.^28^, ^29^, ^30^ Herein, we discovered a novel mechanism underlying the HClnc1 regulation of PKM2 activity, showing that regulating PKM2 activity is intricate and difficult. Further research is needed to determine whether or not HClnc1 affects PKM2's subcellular position, and attempts should be made to determine whether the underlined regulatory mechanisms “cross‐talk.”

As a critical mediator of the Warburg effect in tumor growth, PKM2 is important for HCC cell proliferation and tumor progression.^29^, ^34^ However, its regulation in HCC cells is still poorly understood. Our results determined that an lncRNA overexpressed in HCC stabilizes PKM2 to promote both cytoplasmic aerobic glycolysis and nuclear STAT3 phosphorylation. Although we could not rule out other HClnc1 effector genes, the current data identified PKM2 as a critical target of HClnc1 in HCC development and progression.

## 5 CONCLUSION

Altogether, our obtained data shed light on the mechanisms behind HCC progression. HClnc1 plays a vital role in regulating the Warburg effect and PKM2/STAT3 signaling cascades, giving the first indication of an HClnc1/PKM2 signaling in HCC (Figure 7H). HClnc1/PKM2 signaling cascades may have translational value, given that it can potentially serve as a prognostic predictor and a therapeutic target for HCC. To implement HClnc1 in HCC treatment, extensive research is needed to pinpoint the particular locations that mediate the interaction between HClnc1 and PKM2, which is significantly associated with the development of specific inhibitors targeting HClnc1/PKM2 signaling.

## AUTHOR CONTRIBUTIONS

**Qian Zhu:** Conceptualization (lead); data curation (lead); software (lead); writing – original draft (lead). **Zhengqing Lei:** Resources (supporting); software (supporting). **Chang Xu:** Data curation (supporting). **Zheng Zhang:** Data curation (supporting); resources (supporting). **Zeqian Yu:** Data curation (supporting); resources (supporting). **Zhangjun Cheng:** Data curation (supporting); methodology (supporting). **Pengfeng Xiao:** Validation (supporting); writing – review and editing (equal). **Shufeng Li:** Supervision (supporting); writing – review and editing (equal). **Weiping Yu:** Supervision (supporting); writing – review and editing (equal). **Jiahua Zhou:** Conceptualization (equal); supervision (lead); writing – review and editing (lead).

## FUNDING INFORMATION

This work was supported by the National Natural Science Foundation of China (81572408 and 81071967), the Science and Technology Planning Project of Jiangsu Province (No. BE2015712), the Program of Medical Innovation Team and Leading Medical Talents in Jiangsu Province (2017ZXKJQW09), the Postgraduate Research & Practice Innovation Program of Jiangsu Province (KYCX17_0181) and the Natural Science Foundation of Hubei Province (2018CFB783).

## CONFLICT OF INTEREST STATEMENT

The authors declare that they have no competing interests.

## ETHICAL APPROVAL AND CONSENT TO PARTICIPATE

Eastern Hepatobiliary Surgery Hospital's Institutional Review Board gave their approval to the project. Animal experiments were carried out in accordance with the Health Guide for the Care and Use of Laboratory Animals, which was authorized by the Eastern Hepatobiliary Surgery Hospital's Animal Experimental Research Ethics Committee.

## PATIENT CONSENT FOR PUBLICATION

Not applicable.

## Supporting information


**Data S1:** Supporting Information

## Data Availability

The microarray data addressed in this research has been submitted to the NCBI GeneExpression Omnibus and can be found under GEO Series entry number GSE112613. Onreasonable request, the corresponding authors will provide all the data supporting the currentstudy's findings.
