# Supplementary material for: LncRNA HClnc1 facilitates hepatocellular carcinoma progression by regulating PKM2 signaling and indicates poor survival outcome after hepatectomy
Source: Cancer Med. 2023 May 22;12(13):14526–44. doi: 10.1002/cam4.6117 (PMC10358232; doi:10.1002/cam4.6117)
Supplement: Supplementary file 1 — Data S1: Supporting Information [file CAM4-12-14526-s001.docx]

**Appendix 1**

Five HCC and paired noncancerous tissue samples were collected for lncRNA-microarray analysis. Another 60 (cohort 1) and 120 (cohort 3) pairs of HCC and adjacent tissues samples were used for validation of the microarray data by quantitative real-time PCR (qRT-PCR) and survival analyses, respectively. The expression level of HClnc1 in tissues was evaluated by ISH using a specific digoxin-labelled HClnc1 probe on tissue arrays containing 80 pairs (cohort 2) of HCC and adjacent tissues samples. 238 HCC tissues (cohort 4) were synchronous detected the expression of HClnc1 and PKM2. In addition, the clinicopathologic characteristics were compared pairwise to demonstrate the balance of baseline between any two cohorts.

**Microarray and Computational Analysis.**

Briefly, samples (five HBV-related HCC tissues and five corresponding nontumor tissues; Supporting Table 1) were used to synthesize double-stranded complementary DNA (cDNA), and double-stranded cDNA was labeled and hybridized to the 12ⅹ135K LncRNA Expression Microarray (Arraystar, Rockville, MD). After hybridization and washing, processed slides were scanned with the Axon GenePix 4000B microarray scanner (Molecular Devices, Sunnyvale, CA). Raw data were extracted as pair files using NimbleScan software (version 2.5; Roche NimbleGen, Inc., Madison, WI). NimbleScan software’s implementation of RMA offers quantile normalization and background correction. Differentially expressed genes were identified through the random variance model.16 A P value was calculated using the paired t-test. The threshold set for up- and down-regulated genes was a fold change >¼ 2.0 and a P value <¼ 0.05.

**Cell lines and tumor samples**

All cell lines including HCCLM3, SMMC7721, LO2, Hep3B, HepG2, and Huh7 were purchased from Cell Bank of Type Culture Collection of Chinese Academy of Sciences. All cell lines were cultured at 37°C in an atmosphere containing 5% CO_2_ in Dulbecco’s Modified Eagle’s Medium (DMEM) supplemented with 10% fetal bovine serum. We collected 60 HCC tissues and paired non-cancerous tissue samples for microarray analysis.

Only individuals with a confirmed pathological HCC diagnosis and no preceding neoadjuvant chemotherapy or radiotherapy were included in the study. Patients who died from non-hepatic diseases or accidents, as well as those who lacked comprehensive clinical information and laboratory testing, were excluded from the study. The patients were followed up until November 15, 2017, and the patients who were alive at the end of the study period were censored. The approval for this study was provided by the Clinical Research Ethics Committees of the involved organizations. The research was carried out in conformity with the provisions of the 1975 Helsinki Declaration.

**Cell treatment**

The small interfering RNAs (siRNAs, 50 nM) against human lncRNA (HClnc1 and NR_125715) and PKM2 were transfected into the HCC cells using the lipofactamin2000 (invitrogen, CA) according to the manufacturer’s instructions, while nonspecific siRNA was used as negative controls. The sequences of HClnc1 siRNAs/shRNAs were from the cDNA region of HClnc1 with no overlap with the *PKM2* gene. One set of the siRNAs and shRNAs targets the HClnc1 cDNA region (5’-GCUCUGUGUCACUAUGUACTT-3’) while the other set targets the HClnc1 DNA region (5’-CCUGUCAAAUGCAGGCCAUTT-3’). All the HClnc1 siRNAs and shRNAs cannot bind with the three transcript variants of the *PKM2* gene. All the siRNAs were purchased from GenePharma Company (Shanghai, China). The plasmids encoding human lncRNA (HClnc1, GenBank accession number ENST00000603052) were transfected into the HCC cells using the lipofactamin2000 (invitrogen, CA), while nonspecific plasmid was used as negative controls.

**Cell viability assay**

Cell viability assays were performed using the Cell Counting Kit 8 (CCK-8, Donjindo). Briefly, control and treated HCC cells were seeded into 96-well plates at an initial density of 3.0×10^4^ cells per 100μl. After 24, 48, 72, and 96 hours of cultivation, CCK-8 solution (10μl per 100μl of medium in each well) was added to each well and incubated for 2 h. The spectrophotometric absorbance was measured using a microplate reader (Synergy HT, Bio-Tek) at 450 nm. Based these readings, the percentage of surviving cells in each treated group was plotted. Six replicates for each group and the experiment were carried out with at least 3 times.

**Flow cytometric analysis**

HCC cells were plated at a concentration of 6×10^5^ per well and fixed with chilled 70% ethanol overnight at 4°C. Subsequently, we labeled single-cell suspensions in phosphate-buffered saline with 400μl PI (Sigma, St Louis, MO), 100μl RNase A and analyzed approximately 6×10^5^ cells in 1ml by flow cytometry (FACSCalibur, BD Biosciences, San Jose, CA, USA). Data were expressed as percentage distribution of cells in G0/G1, S and G2/M phases of the cell cycle.

Apoptosis was also determined by flow cytometry analysis using the Annexin V-PI detection kit. After transfection, cells were harvested for Annexin V-PI staining according to the manufacturer’s instructions (BD Biosciences PharMingen). The double-stained cells were analyzed by flow cytometry, and the early or late apoptotic cells were measured.

**Immunohistochemical staining**

The expressions of PKM2, PKM1, CD34 and Ki67 were examined with primary antibodies (PKM2, 1:1000; PKM1, 1:600; CD34, 1:100; Ki67, 1:100) from Abcam according to the manufacturer’s instructions. The tissue slides were examined independently by 2 investigators blinded to both the clinical and pathologic data. Protein expression was quantified using a visual grading system based on the extent of staining (percentage of positive tumor cells on a scale of 0–4: 0, none; 1, 1%–25%; 2, 26%–50%; 3, 51%–75%; 4, >75%) and the intensity of staining (graded on a scale of 0–3: 0, no staining; 1, weak staining; 2, moderate staining; 3, strong staining). For further analysis, the product of the extent and intensity grades was used to define the cutoff value for higher protein expression. Therefore, protein expression was thus classified into 2 categories: high level (grades 4–12) and low level (grades 0–3). The micro-vessel density (MVD) of the tumor tissues was evaluated by staining for CD34. Any discrete cluster or single cell that stainedpositively for CD34 was counted as one microvessel. Paraffin-embedded tissue sections were used for an IHC analysis. A mouse antibody against human CD34 (Santa-Cruz, CA) was applied to the clinical samples, and a rat antibody against mouse CD34 was used to detect the MVD of the

subcutaneous tumor tissues. The CD34 expression was evaluated under a light microscope at a 400× magnification. All discrepancies in scoring were reviewed until a consensus was reached. Two investigators whowere blind to the experimental design quantified the MVD.

**Western blotting**

For further validation of the RNA pull-down assay results in Huh7 cells and fresh HCC tissue, streptavidin-agarose beads were added to each binding reaction, washed and then boiled in SDS buffer at 100°C for 10 min. An anti-GAPDH antibody (Sigma-Aldrich) was used as the loading control. Anti-PKM2 was diluted 1:1000 (Abcam, USA), anti-PKM1 was diluted 1:600 (Cell Signaling Technology, Boston, USA), anti-CD34 was diluted 1:1000 (Abcam, USA), and anti-Ki67 and anti-EZH2 were diluted 1:100 and 1:5000, respectively (Abcam, USA).

**Follow-up**

Patient follow-up was performed every 2-3 months during the first year after surgery and 3-6 months thereafter until November 15, 2017. The median follow-up duration was 46 months (range 6–104 months). All follow-up examinations were performed by two physicians blinded to study data. All patients were monitored by abdomen ultrasonography, chest X-ray, and measurement of serum AFP every month during the first year after surgery and every 3-6 months thereafter. A computed tomography (CT) scan or magnetic resonance imaging (MRI) of the abdomen was performed every 6 months or immediately after a recurrence was suspected. Diagnostic criteria for recurrences were equal to that for preoperative diagnosis. Once recurrent tumors were confirmed, treatment was implemented based on tumor diameter, number, location, and vessel-invasion and hepatic function data. Recurrence-free survival (RFS) was calculated from the date of tumor resection until tumor recurrence or the last observation. Overall survival (OS) was defined as the length of time between surgery and death or the last follow-up examination.

**Dataset**

LncRNAs were characterized from the gene expression data. Our lncRNA microarray analysis of the five HCC tissues and [paired normal tumor-adjacent liver tissues](javascript:;) was uploaded to the Gene Expression Omnibus (GEO) database, and the dataset accession number is GSE112613. LncRNA HClnc1 was validated from the GEO repository (GSE45436, GSE55092, and GSE62232). The details of the bioinformatics analyses are provided in the Appendix 1.

**Quantitative real-time PCR (qRT-PCR)**

Real-time quantitative PCR was performed on triplicate samples in a reaction mix of SYBR Green (Roche, USA) with LightCycler96 Real-Time PCR System (Roche, USA). β-actin expression was used as an internal control. The sequences of primers and probes used for qRT-PCR in the study were presented in Table SII. The relative mRNA levels were calculated based on the Ct values and normalized using the β-actin expression. The relative expression of RNAs was calculated using the comparative Ct method and presented as mean ± SD.

**5’and 3’rapid amplification of cDNA ends (RACE) analysis**

We used the 5’- and 3’-RACE analyses to determine the transcriptional initiation and termination site of HClnc1 using a SMARTer RACE cDNA Amplification Kit (Clontech, Palo Alto, CA, USA), according to the manufacturer’s instructions. Polymerase chain reaction (PCR) of the internal region was performed when starting points of 5’ and 3’ RACE had an unamplified gap. RACE PCR products were separated on a 1% agarose gel. Gel products were extracted with the Gel and PCR Clean-Up System (Clare Chemical Research, A9282), cloned into the pGEM-TVector Systems I (Promega, A3600) and sequenced bidirectionally using the M13 forward and reverse primers by Sanger sequencing at ABI (3730). At least five colonies were sequenced for every RACE PCR product that was gel purified. The gene-specific primers used for the PCR of the RACE analysis were given at Table SII.

**Bioinformatics Analysis**

Human exon arrays for hepatocellular carcinoma (HCC) and normal adjacent tissues were downloaded from the NCBIs Gene Expression Omnibus (GEO, http://www.ncbi.nlm.nih.gov/geo/). The datasets GSE45436, GSE55092 and GSE62232 consisted of 227 paired HCC tissues and adjacent tissues. To gain further insight into the biological pathways involved in HCC pathogenesis through lncRNA HClnc1, the gene set enrichment analysis (GSEA) was performed. The gene sets showing FDR, 0.25, a well-established cut-off for the identification of biologically relevant genes, were considered enriched between the classes under comparison. The gene sets collection (c2.all.v4.0.symbols.gmt) from the Molecular Signatures Database–MsigDB (http://www.broad.mit.edu/gsea/msigdb/index.jsp) was used for the enrichment analysis. PhyloCSF (1) was used to assess coding potential in the transcripts based on evolutionary signatures in the 29-manmanlian genome alignment. Prediction error curves (PEC) (2) were used to assess and compare predictions in survival analysis. The PEC curves were plotted by R package "PEC".

**Expression profile analysis of lncRNAs**

The Arraystar Human LncRNA Microarray V4.0 (8×60K; ArrayStar), containing 16517 probes specific for human long noncoding RNAs splicing sites, was used to proﬁle lncRNA expression. After hybridization and washing with samples, 5 paired liver cancer tissues and matched adjacent normal tissues were analyzed on the lncRNAs chips. Exogenous RNAs developed by External RNA Controls Consortium were used as controls. In total, 40,173 lncRNAs and 20,730 coding transcripts were collected from the most authoritative databases, such as RefSeq, the UCSC Known Genes dataset, Gencode, Ensembl, and RNAdb 2.0, as described in the manufacturer’s instructions, and the raw data can be accessed via GSE112613.

**RNA-protein interaction proteomics**

Re-suspend cells with pre-cooling PBS buffer, crosslink with 3% formaldehyde at room temperature on an end-to-end shaker for 10min. Quench crosslinking with 125 mM glycine for 5min, spin at 1000RCF for 3 min and discard supernatant, wash cell pellets twice with cooling PBS. For each 2×10^7^ cells, add 1mL Lysis buffer, sonicate cell lysate in an ice-water bath and check every 10min until the cell lysate is no longer turbid. Spin at top speed, transfer supernatant to 2 volume of Hybridization Buffer, mix well and incubate at 37 ℃. Pre-binding probes (6 for target RNA, 1 for NC and PC, 100pmol per 2×10^7^ cells) to streptavidin beads for 30min, wash out unbinding probe, and mix with cell lysate, hybridize at 37 ℃ overnight on an end-to-end shaker. Wash beads 5 times with 1mL pre-warming Wash Buffer, 5min per washing. At the last washing step, transfer 1/20 beads for qPCR analysis. Add 100μL Elution Buffer, 20U Benzonase, elute protein at 37 ℃ for 1h. Transfer supernatant to new low binding eppendorf tube. Wash beads with 100μL Elution buffer once, and combine 2 supernatants. Reverse cross-linked sample at 95 ℃, 30 min precipitate protein with 0.1% SDC and 10% TCA at 4 ℃ for 2h. Spin at top speed, wash pellets with pre-cold 80% acetone 3 times.

**Liquid chromatography-mass spectrometry (LC-MS) analysis, database search, and protein identification**

***Peptide desalting for LC-MS/MS***

Equilibrate C_18_ column with 200 μL CAN; Wash out ACN with 200 μL 0.1% FA 2 times, discard the wash out; Load peptide solution to C_18_ tip column, let the solution flow through the column slowly, and collect flow through (A); Repeat the peptide loading step once; Wash column with 200 μL 0.1% FA, discard the wash out; Elute peptide with 50 μL 70% ACN, collect elution (B) with new ep; Repeat the desalting step (up 6 steps) once more with flow through (A); Merge 2 elution (B) , vacuum dry the elution under 4 ℃ or RT; Re-suspend peptide with 10μL 0.1% FA for LC-MS/MS analysis, or store peptide powder at -80 ℃.

***LC-MS/MS***

For each sample, 1/2 peptide was separated and analyzed with a Nano-HPLC (EASY-nLC1200) coupled to Q-Exactive mass spectrometry (Thermo Finnigan). Separation was performed using a reversed-phase column (100 μm, ID × 15 cm, Reprosil-Pur 120 C18-AQ, 1.9um, Dr. Math). Mobile phases were H_2_O with 0.1 % FA, 2 % ACN (phase A) and 80 % ACN, 0.1 % FA (phase B). Separation of sample was executed with a 120 min gradient at 300 nL/min flow rate. Gradient B: 8 – 35 % for 92 min, 35 – 45 % for 20 min, 45 - 100 % for 2 min, 100 % for 2 min, 100 – 2 % for 2 min and 2 % for 2 min.

Data dependent acquisition was performed in profile and positive mode with Orbitrap analyzer at a resolution of 70,000 (200 m/z) and m/z range of 350 – 1400 for MS1; For MS2, the resolution was set to 17,500 (200 m/z). The automatic gain control (AGC) target for MS1 was set to 1.0e+06, and 1.0e+05 for MS2. The top 10 most intense ions were fragmented by HCD with normalized collision energy (NCE) of 28 %, and isolation window of 2 m/z. The dynamic exclusion time window was 30 s.

***MaxQuant database search***

Raw MS files were processed with MaxQuant (Version 1.5.6.0). The human protein sequence database (Uniprot_HUMAN_2016_09) was downloaded from UNIPROT. This database and its reverse decoy were then searched against by MaxQuant software. The quantification type was MS1; Trypsin was set as specific enzyme with up to 2 miss cleavage; Oxidation [M] and Acetyl [protein N-term] were considered as variable modification, Carbamidomethyl [C] was set as fixed modification; Both peptide and protein FDR should be less than 0.01. Only unmodified unique peptides were used for quantification. The iBAQ label free quantification was also measured with log fit checked.

***In vivo* experiments**

A power analysis (GPower3.1.3; a = 0.05, power= 0.80; effect size calculation based on estimated effect sizes in tumor weight of treatment and control group) (3) was used to determine adequate statistical power. Mice were monitored for tumor growth by researchers blinded to the study design.

In order to clarify the effect of HClnc1 *in vivo*, 4-week-old male BALB/c nude mice obtained from Experimental Animal Centre of Nanjing University were used in our study. Huh7 cells (1.0×10^7^ or 5×10^6^ cells for establishing HClnc1-overexpressing with PKM2 inhibition HCC xenograft model), LM3 cells (1.0×10^7^) and SMMC7721 cells (1×10^7^) were injected subcutaneously into the right flank of these mice to establish the gastric cancer xenograft model. Two weeks after subcutaneous inoculation, mice were randomly divided into different groups and were injected with PBS, control shRNA, HClnc1 shRNA1, HClnc1 shRNA2, PKM2 shRNA, or HClnc1 overexpression virus by ways of multipoint intratumoral injection every other day for fourteen days. Tumor volume (mm^3^) was estimated by the formula: tumor volume (mm^3^) = longer diameter × shorter diameter^2^ ×1/2.

To further investigate the effect of HClnc1 on tumor invasion *in vivo*, we developed a HCC metastasis model in nude mice. The Huh7 cells (5 × 10^6^), LM3 cells (5× 10^6^) and SMMC7721 cells (5×10^6^) were subcutaneously injected through the right flank of 4-week-old BALB/c nude mice. Mice were inspected daily. Tumors were measured with a caliper every 4 days. Tumor volume (mm^3^) was calculated by the formula: tumor volume (mm^3^) = shorter diameter2 × longer diameter/2. Two weeks after tumor inoculation, the tumor volume was ∼30 mm^3^. The animals were randomly divided into different groups with intratumoral injection of PBS, adenovirus encoding a control shRNA, or HClnc1 shRNA1, or HClnc1 shRNA2 or HClnc1 (1×10^7^ tumor/mouse, twice a week) for 13 weeks. For lung metastasis examination, mice were sacrificed at week 13. The numbers of lung metastatic foci were determined in H&E stained lung tissue sections under a binocular microscope (Leica, DM 300). All experimental procedures were approved by the Institutional Animal Care and Use Committee of School of Medicine, Southeast University.

**RNA pull-down assay**

The biotin-labeled lncRNA-HClnc1 and the antisense RNA were in vitro transcribed with a Biotin RNA Labeling Mix (Roche) and the T7 RNA polymerase (Roche), treated with RNase-free DNase I (Roche) and purified with an RNeasy Mini Kit (QIAGEN). One milligram of protein from Huh7 cell extracts was then mixed with 50 pmol of biotinylated RNA. Sixty microliters of washed streptavidin agarose beads (Invitrogen, USA) was then added to each binding reaction and washed. The associated proteins were resolved by SDS-PAGE, and specific bands were excised and analyzed by mass spectrometry.

**RNA immunoprecipitation**

RNA immunoprecipitation (RIP) experiments were performed using a Magna RIP™ RNA-Binding Protein Immunoprecipitation Kit (Millipore, USA) according to the manufacturer’s instructions. Antibody for RIP assays of PKM2 and EZH2 (Abcam, USA) were diluted as 1:200 and 1:500.

**The ELISA assay**

The PKM2 ELISA assay was performed using a PKM2 quantitative kit (USCN, USA) according to the manufacturer’s instructions. The serum samples of the patients were analyzed at a 1:20 dilution defined by the pre-tests and manufacturer’s instructions. The supernatant of the Huh7 cells was prepared 120 h after transfection with the Lv-lncRNA-HClnc1 or Lv-NC vectors.

**Statistical analyses**

Data from least 3 independent experiments performed in triplicates are presented as the means ± standard error (SE) or standard deviation (SD). Error bars in the scatter plots and the bar graphs represent SE or SD. Date were examined whether they were normally distributed with the One-Sample Kolmogorov-Smirnor test. If the data were normally distributed and the variation between groups were comparable, the comparisons of measurement data between two groups were performed using the paired-sample t test or independent-sample t test. The comparisons among three or more groups were firstly performed by One-Way ANOVA test if the variation between groups were comparable. If the results showed significant difference, the Student Newman Keuls analysis was used to test the difference between the two groups. When the data were shown the skewed distribution, comparisons were performed by nonparametric tests. Enumeration data were examined by Chi-square test or Fisher Exact test. The recurrence-free survival (RFS) and overall survival (OS) were considered to be the primary endpoints. We defined the recurrence-free survival or overall survival as the time interval between the date of diagnosis to the first HCC recurrence (local or distant) or to the subject’s death from HCC. Overall survival was evaluated by the Kaplan–Meier survival curve and the Log-rank test. The Cox proportional hazards model was used to determine the independent factors, which were based on the variables selected by a univariate analysis. We also formally examined for effect modification by HClnc1 by testing the statistical significance of HClnc1×TNM stage interaction terms in multivariable-adjusted Cox models using a postestimation Wald test to obtain an omnibus P value for interaction between HClnc1 and TNM stage. The correlation of the two genes was examined by Spearman correlation test. To generate the ROC curves, patients were classified as surviving either longer or shorter than the median OS, excluding patients who were alive for durations less than the median OS at last follow-up. Statistical tests and P-values were two-sided. Differences were considered significant with a value of P < 0.05.

All statistical analyses were carried out using the program R (www.r-project.org) or SPSS for Windows 16.0.0 software (SPSS Inc., Chicago, IL, USA).

**References**

1. Lin MF, Jungreis I and Kellis M: PhyloCSF: a comparative genomics method to distinguish protein coding and non-coding regions. Bioinformatics 27: i275–i282, 2011.

2. Mogensen UB, Ishwaran H and Gerds TA: Evaluating random forests for survival analysis using prediction error curves. J Stat Soft 50: 1–23, 2012.

3. Hu Y, Wang J, Qian J, Kong X, Tang J, Wang Y, Chen H, Hong J, Zou W, Chen Y, *et al*: Long noncoding RNA GAPLINC regulates CD44-dependent cell invasiveness and associates with poor prognosis of gastric cancer. Cancer Res 74: 6890–6902, 2014.

4. Shevchenko A, Tomas H, Havlis J, Olsen JV and Mann M: In-gel digestion for mass spectrometric characterization of proteins and proteomes. Nat Protoc 1: 2856–2860, 2006.

5. Cox J and Mann M: MaxQuant enables high peptide identification rates, individualized ppb-range mass accuracies and proteome-wide protein quantification. Nat Biotechnol 26: 1367–1372, 2008.

6. Cox J, Neuhauser N, Michalski A, Scheltema RA, Olsen JV and Mann M: Andromeda: a peptide search engine integrated into the MaxQuant environment. J Proteome Res 10: 1794–1805, 2011.

7. Trapnell C, Pachter L and Salzberg SL: TopHat: discovering splice junctions with RNA-Seq. Bioinformatics 25: 1105–1111, 2009.

8. Trapnell C, Hendrickson DG, Sauvageau M, Goff L, Rinn JL and Pachter L: Differential analysis of gene regulation at transcript resolution with RNA-seq. Nat Biotechnol 31: 46–53, 2013.

9. Trapnell C, Roberts A, Goff L, Pertea G, Kim D, Kelley DR, Pimentel H, Salzberg SL, Rinn JL and Pachter L: Differential gene and transcript expression analysis of RNA-seq experiments with TopHat and Cufflinks. Nat Protoc 7: 562–578, 2012.

10. Heinz S, Benner C, Spann N, Bertolino E, Lin YC, Laslo P, Cheng JX, Murre C, Singh H and Glass CK: Simple combinations of lineage-determining transcription factors prime cis-regulatory elements required for macrophage and B cell identities. Mol Cell 38: 576–589, 2010.

11. Shen L, Shao N, Liu X and Nestler E: ngs.plot: Quick mining and visualization of next-generation sequencing data by integrating genomic databases. BMC Genomics 15: 284, 2014.

12. Kang J, D'Andrea AD and Kozono D: A DNA repair pathway-focused score for prediction of outcomes in ovarian cancer treated with platinum-based chemotherapy. J Natl Cancer Inst 104: 670–681, 20

**Supplementary figures**

**Figure S1. Clinical relevance of lncRNA candidate ENST00000603052 in HCC.** **(A)** Differential expression of lncRNAs & mRNAs in HCC, the flow chart for selected candidate lncRNAs in 532 up-regulated lncRNAs in HCC tissue is shown. **(B)** Ten candidate lncRNAs expression were quantified in HCC tissues and their adjacent tissues in cohort 1 & cohort 3. *n* = 60, *n* = 120. non-parametric Mann-Whitney test. **(C)** The association between lncRNA candidates and patient survival is shown in 120 patients (cohort 3) with HCC. Log-rank test.

**(D)** The expression of ENST00000603052 was quantified by real-time PCR in total HCC and normal tissues. *n* = 277 (GSE45436, GSE55092, GSE62232). **(E and F)** Survival was analyzed and compared between patients with high and low levels of HClnc1 expression in tumor in cohort 2; n = 80(ISH), log-rank test. **(G)** Multivariable analysis of OS and multivariable analysis of RFS were performed in the cohort 2. All of the bars correspond to 95% confidence intervals. **(H)** The association between lncRNA HClnc1 and patient survival is shown in 154 patients with early HCC. Log-rank test. **(I)** Multivariable analysis of RFS (**F**) and multivariable analysis of OS (**G**) were performed in the cohort 3. All of the bars correspond to 95% confidence intervals. **(J)** The coding potential was analyzed for the HClnc1 sequence across 29 mammals in all 8 reading frames using PhyloCSF. NR_001446 and lncRNA-MVIH served as a control non- coding genes. GAPDH and β-actin served as control coding genes. Scores above 0 suggest coding potential whereas scores below 0 suggest no coding potential. **(K and L)** Coding Potential Calculator software predicted that HClnc1 is an lncRNA. **(M)** HClnc1 has no coding potential. Ribosome profiling relative to whole transcriptome RNA sequencing. x-axis: genomic position at the human GAPDH and the human HClnc1 loci. y-axis: mapped reads. **(N)** Overview of transcription, translation, and biotinylation procedures is shown for HClnc1 *in vitro*. Transcriptional RNA was used as a positive control and no RNA template (Mock) was used as a negative control.

**Figure S2. HClnc1 is an oncogenic lncRNA in HCC.** **(A)** HClnc1 expression was quantified by real-time PCR in different HCC cells. *n* = 3. **(B)** Cell proliferation assay was performed in six HCC cell lines. These six cell lines were classified into three groups according to their proliferation capacities. The high group is HCCLM3 and Huh7 cells (group one), the moderate group is HepG2 cells (group two), and the weak group is SMMC7721 and Hep3B cells (group three). **P <* 0.05, SMMC7721 or Hep3B compared to LO2 cells. ***P <* 0.01, HCCLM3 or Huh7 compared to LO2 cells. n = 3, non-parametric Mann-Whitney test. **(C)** Real-time PCR was performed in Huh7 and LM3 cells after transfection of HClnc1 siRNAs. *n* = 3, non-parametric Mann-Whitney test. **(D)** Real-time PCR was performed in SMMC7721 and Hep3B cells after transfection of HClnc1 overexpressing plasmid. *n* = 3, non-parametric Mann-Whitney test. **(E and F)** Cell proliferation was determined in Huh7 and LM3 cells after control and NR_125715 siRNA1/2 transfection. *n* = 3, non-parametric Mann-Whitney test. **(G)** Representative data of tumors is shown in xenograft model after injection with PBS, control shRNA adenovirus, or HClnc1 shRNA1 adenovirus. Tumor progression was monitored in xenograft model. Tumor volume was monitored and tumor weight was measured after HClnc1 shRNA1/2 adenovirus treatments in the xenograft mouse model. *n* = 5, non- parametric Mann-Whitney test. **(H and I)** IHC staining of Ki67 in the three groups of xenografts is shown. *n* = 8, non-parametric Mann-Whitney test. Error bars in the scatter plots represent SE. **(J and K)** Cell proliferation assay was performed in SMMC7721 and Hep3B cells after HClnc1 overexpression. *n* = 3, non-parametric Mann-Whitney test. **(L and M)** Transwell Matrigel invasion assay was performed in SMMC7721 and Hep3B cells after HClnc1 overexpression. *n* = 3. **(N)** Tumor progression was monitored in xenograft model. Tumors were injected with PBS, control adenovirus, or HClnc1 expressing adenovirus. **(O and P)**Tumor volume (O) was monitored and tumor weight (P) was measured on day 14. *n* =5, non-parametric Mann-Whitney test. **(Q)** Survival analysis was performed in mice bearing HCC cells transfected with HClnc1 overexpression adenovirus, control shRNA adenovirus and PBS. n = 10, Log-rank test. **(R and S)** Representative hematoxylin-eosin staining and summarized data showing tumor lung foci in nude mice at 13 weeks after injection of HClnc1 overexpressing adenovirus. *n* = 10. **(T and U)** Transwell Matrigel invasion assay was performed in LM3 cells transfected with control siRNA, HClnc1 siRNA1/2, and NR_125715 siRNA 1/2, respectively; n = 3. **(V)** Survival analysis was performed in mice bearing HCC cells transfected with HClnc1 shRNA adenovirus, control shRNA adenovirus and PBS. n = 10, Log-rank test. **(W)** Representative hematoxylin-eosin staining and summarized data showing tumor liver foci in nude mice at 13 weeks after injection of HClnc1 shRNA adenovirus. *n* = 8. **P <* 0.05, ***P <* 0.01.

**Figure S3. HClnc1 increase PKM2 mRNA levels.** **P <* 0.05, ***P <* 0.01.

**Figure S4.** GO enrichment analyses in Huh7 cells transfected with si-HClnc1 or control (si-NC)Real-time PCR was performed in SMMC7721 cells after transfected with control vector and HClnc1-overexpression and incubated with CHX. *n* = 5, non-parametric Mann-Whitney test.

**Figure S5. HClnc1 promotes HCC progression via PKM2. (A)** The association between PKM2 and patient recurrence-free survival is shown in 238 patients (cohort 5) with HCC. Log-rank test. **(B and C)** The association between HClnc1 and patient overall and recurrence-free survival are shown in 238 patients (cohort 5) with HCC. Log-rank test. **(D)** Multivariable analysis of RFS was performed in the cohort 5. All of the bars correspond to 95% confidence intervals. **(E)** The expression of the PKM2 in HCC cells or a Negative control (serum) was quantified by ELISA. The data are shown as the means ± standard deviation of three independent biological replicates. The statistical difference was analyzed by Two-sample t-test.


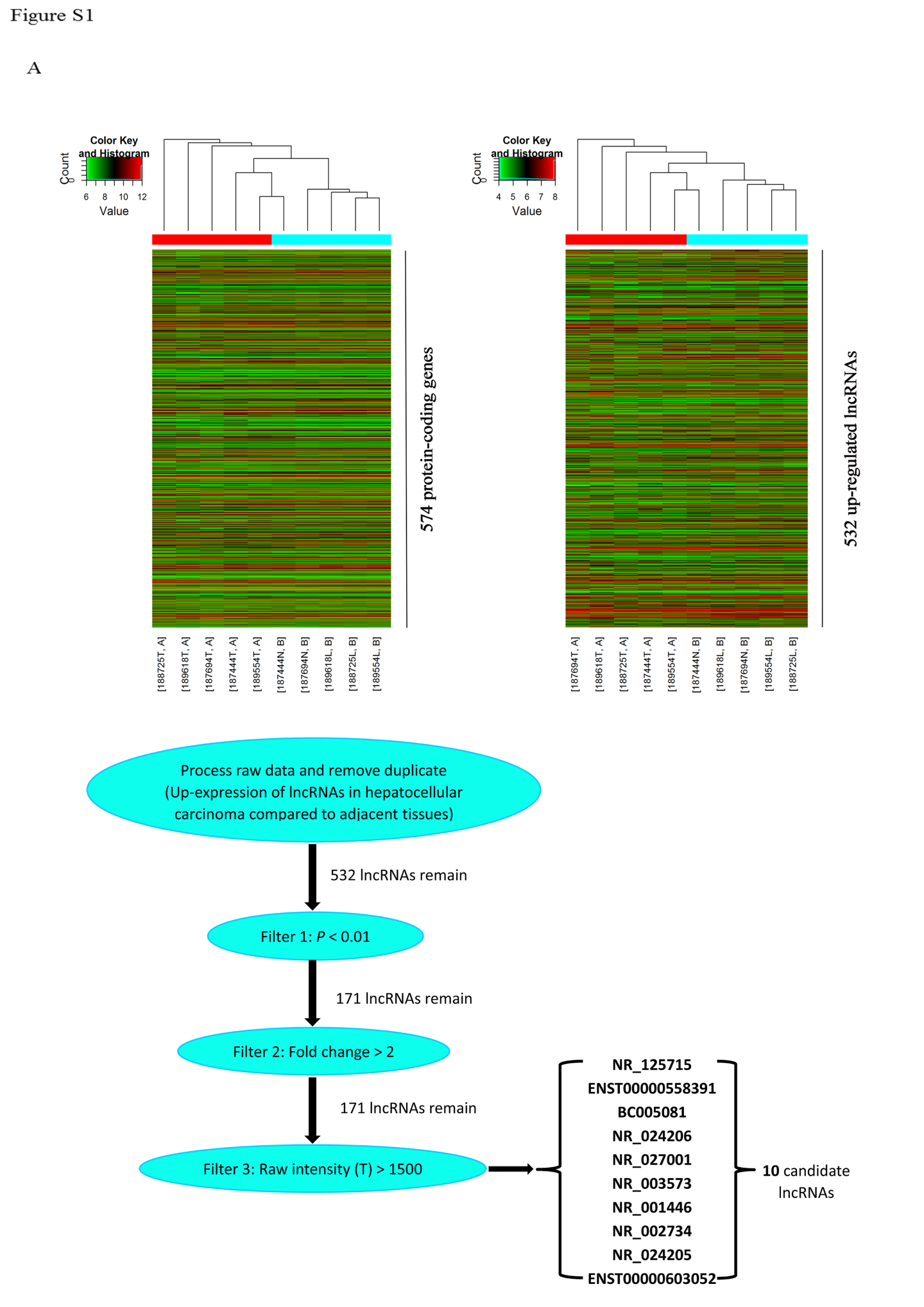


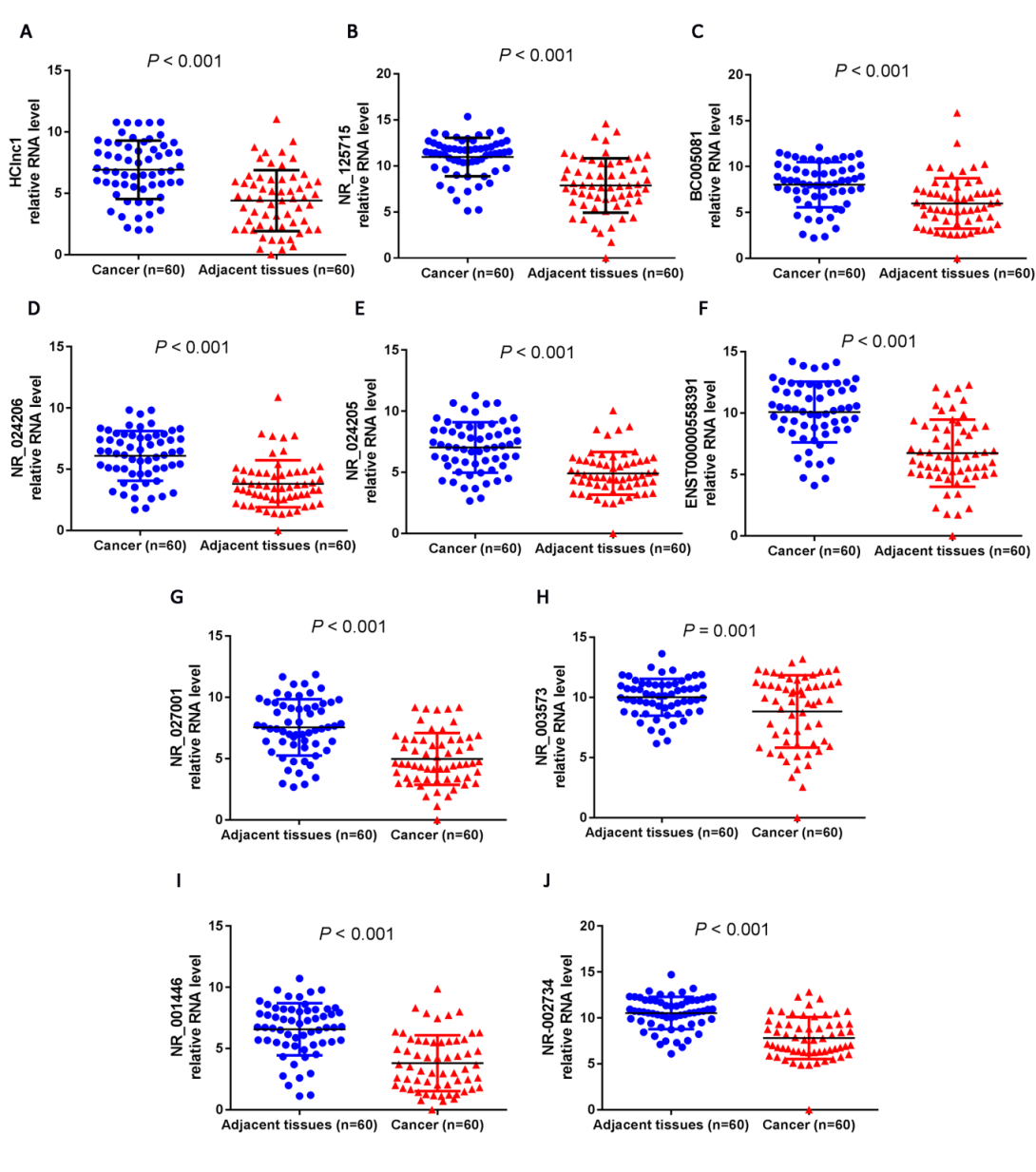


**Figure S1**

**B**


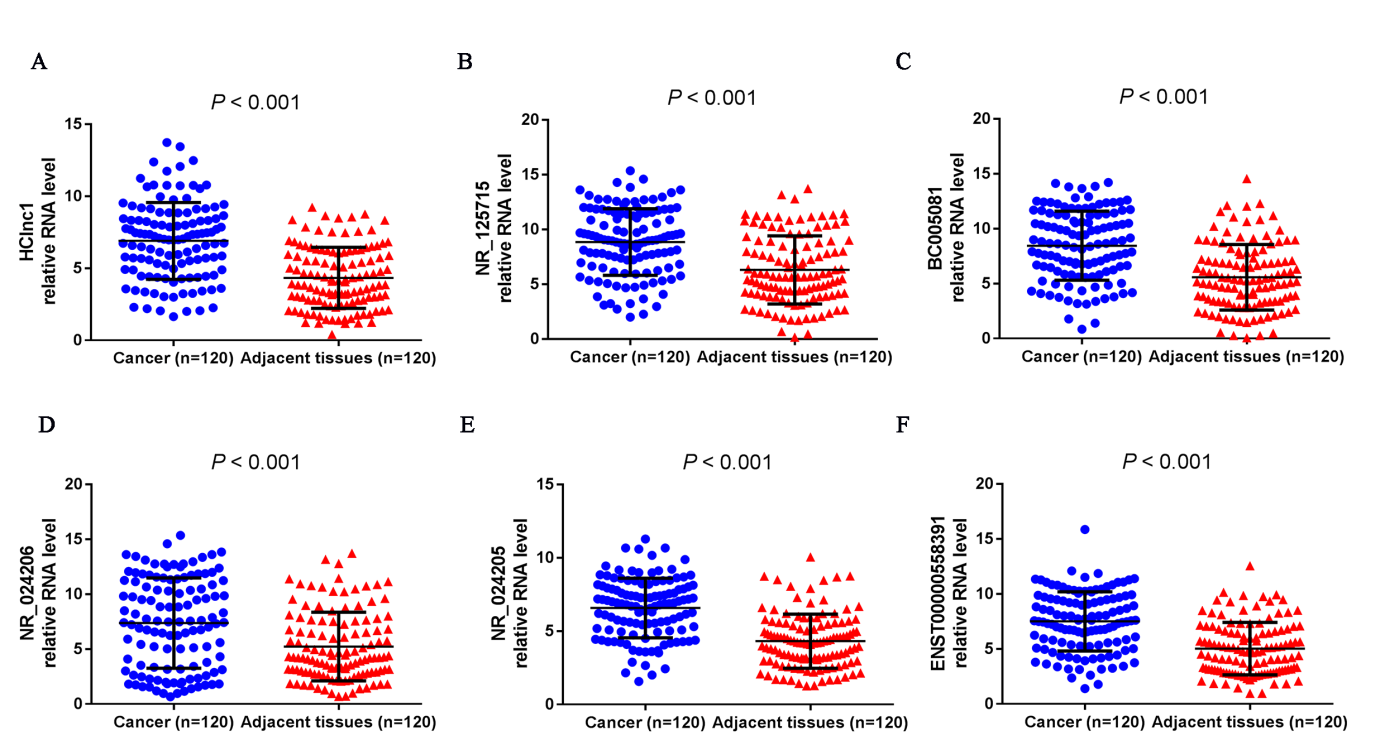


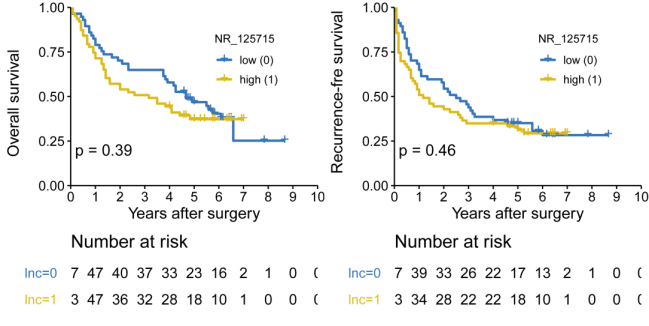

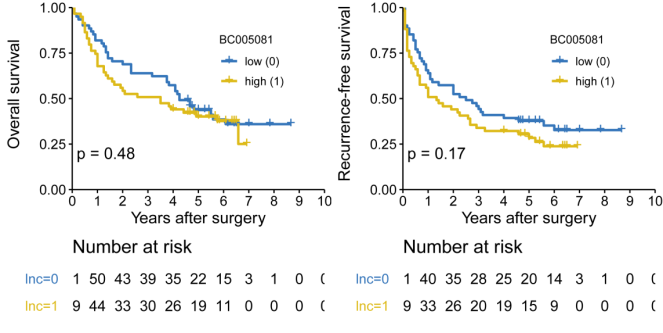


**Figure S1**

**C**


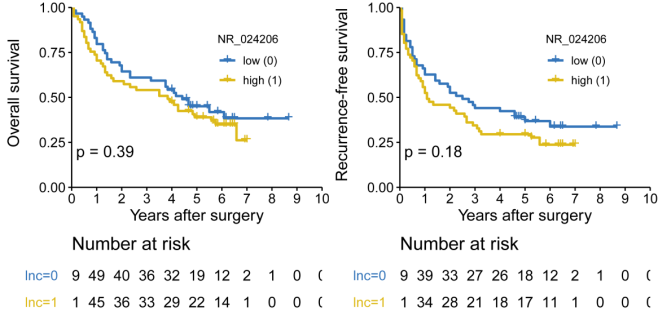

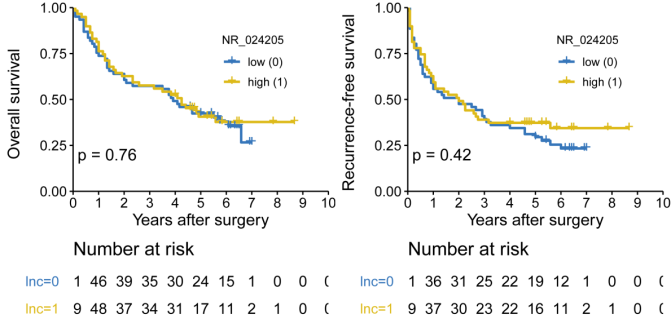


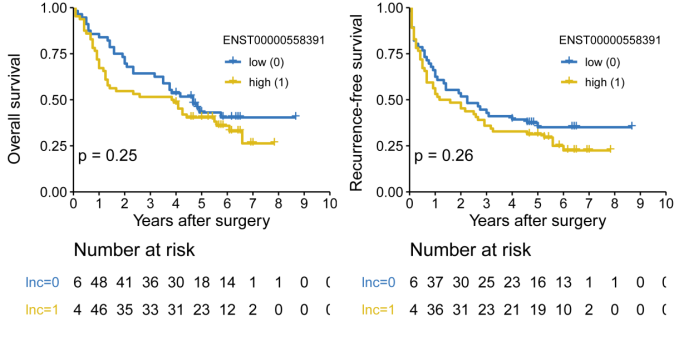


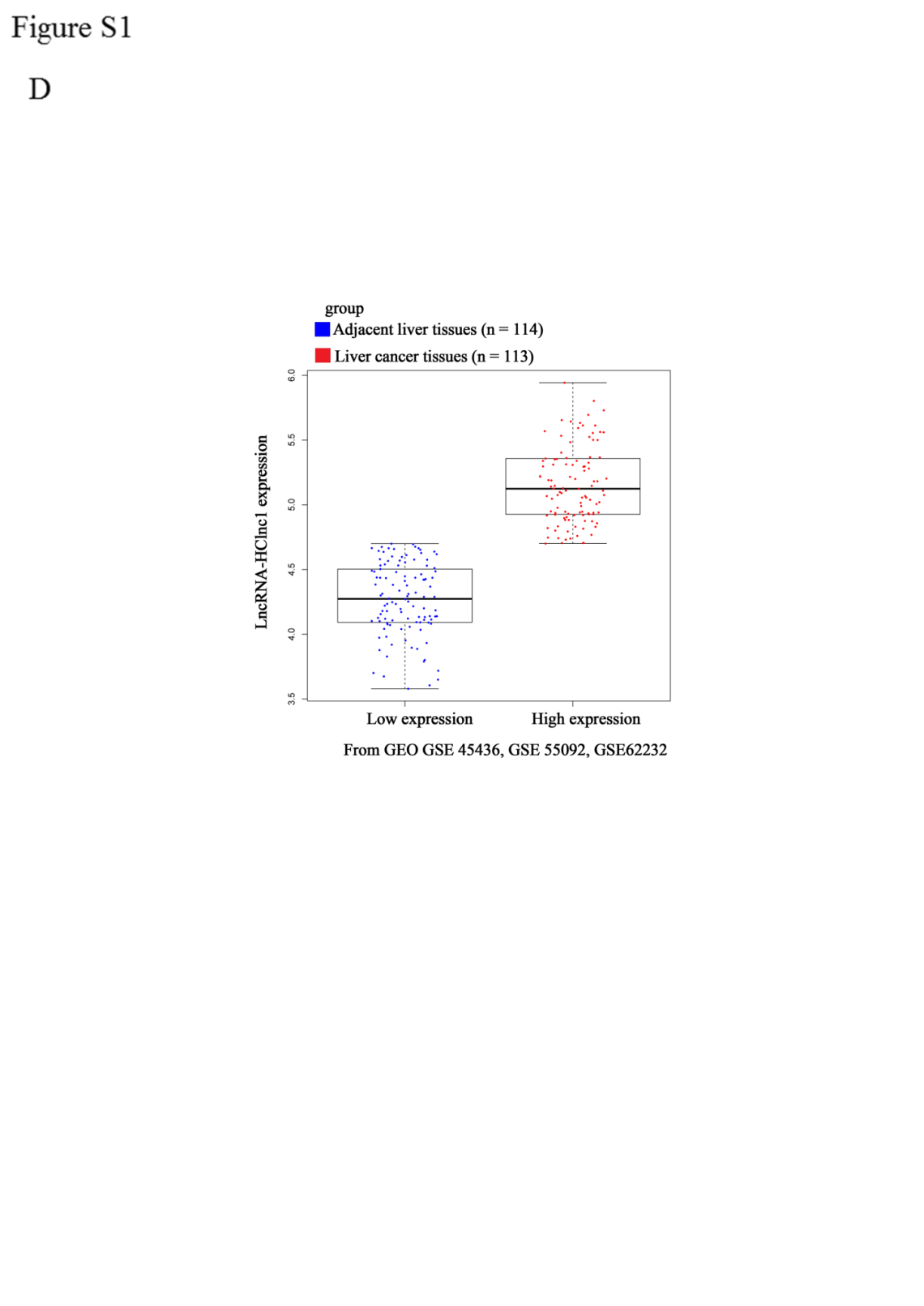


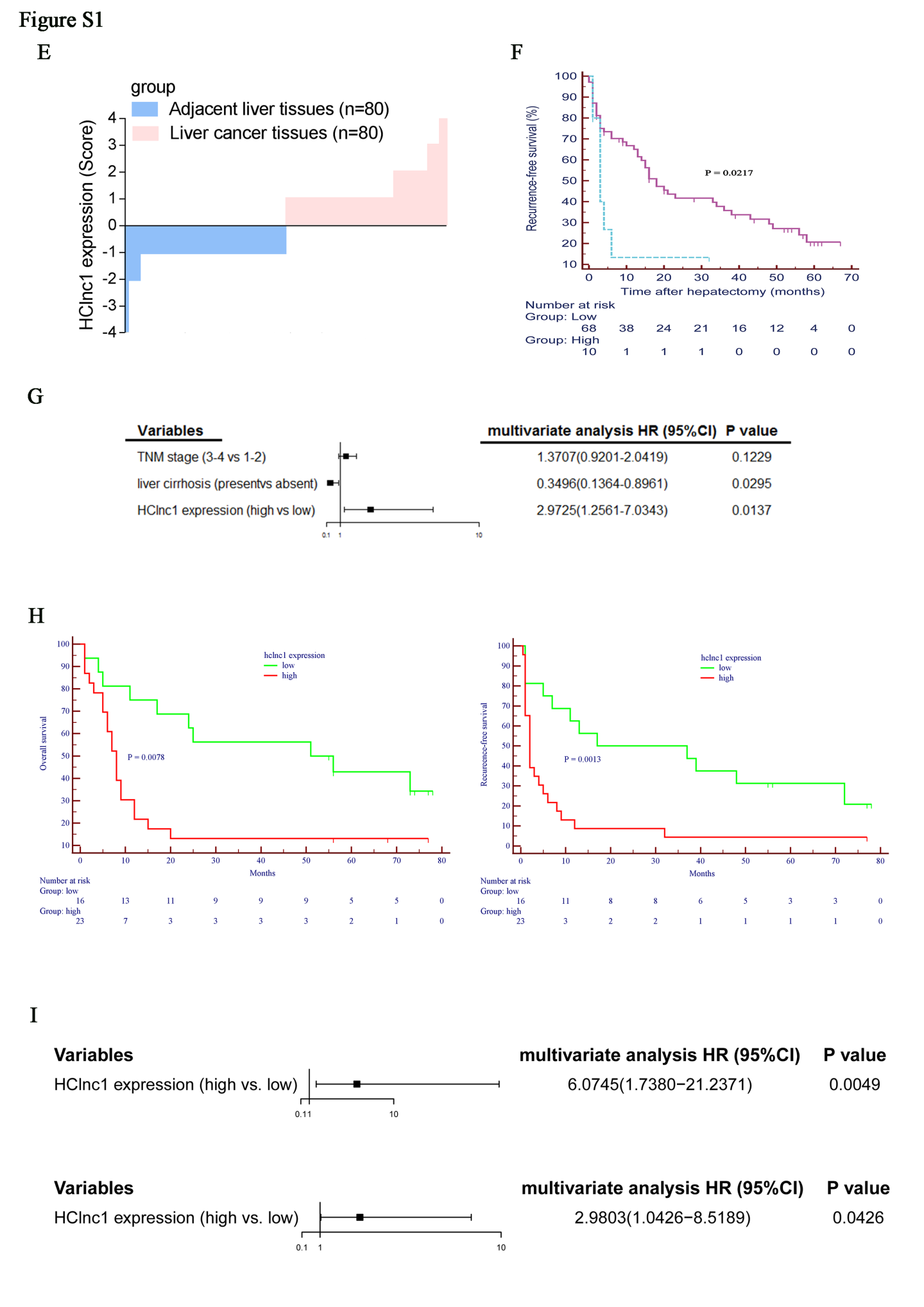


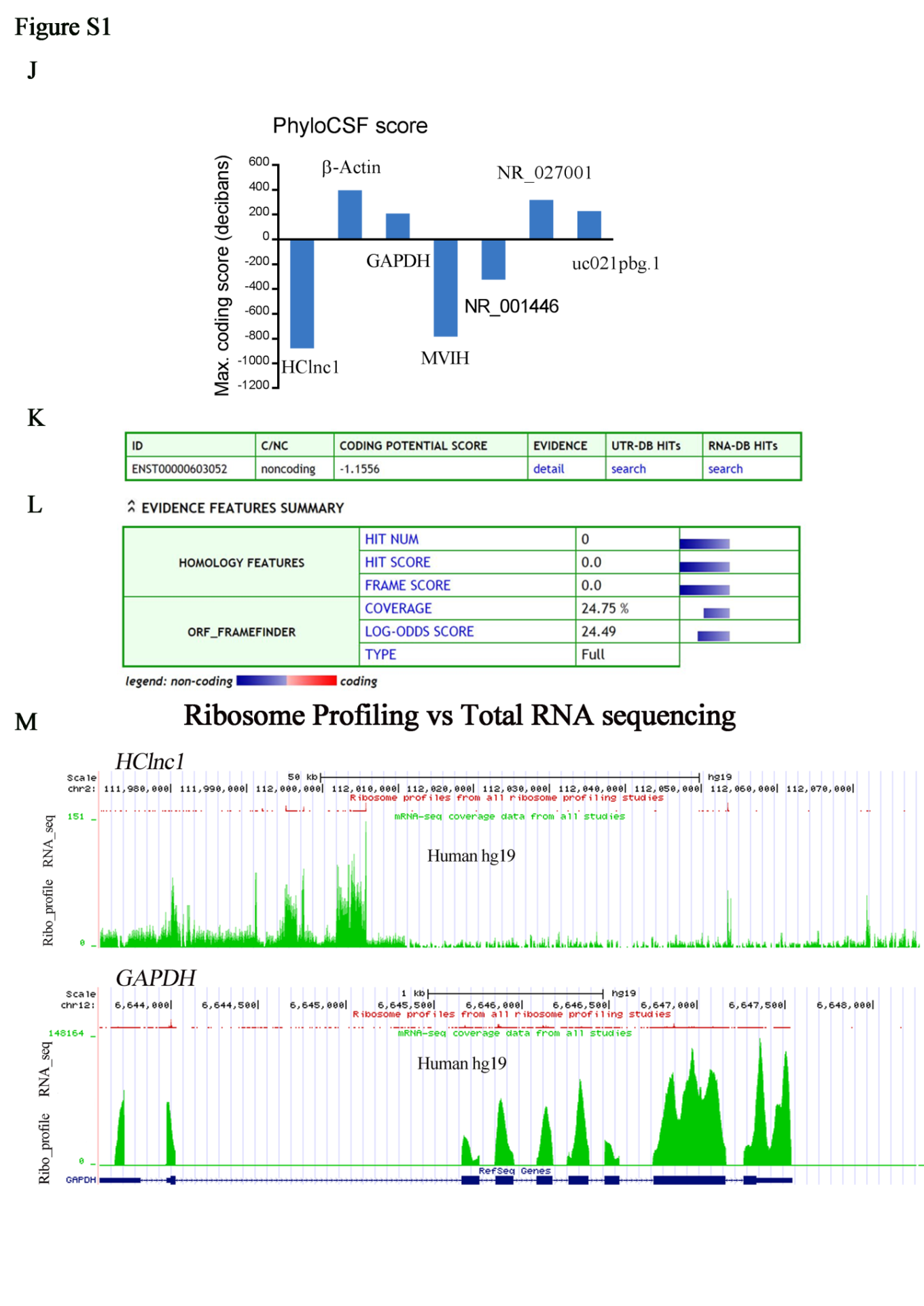


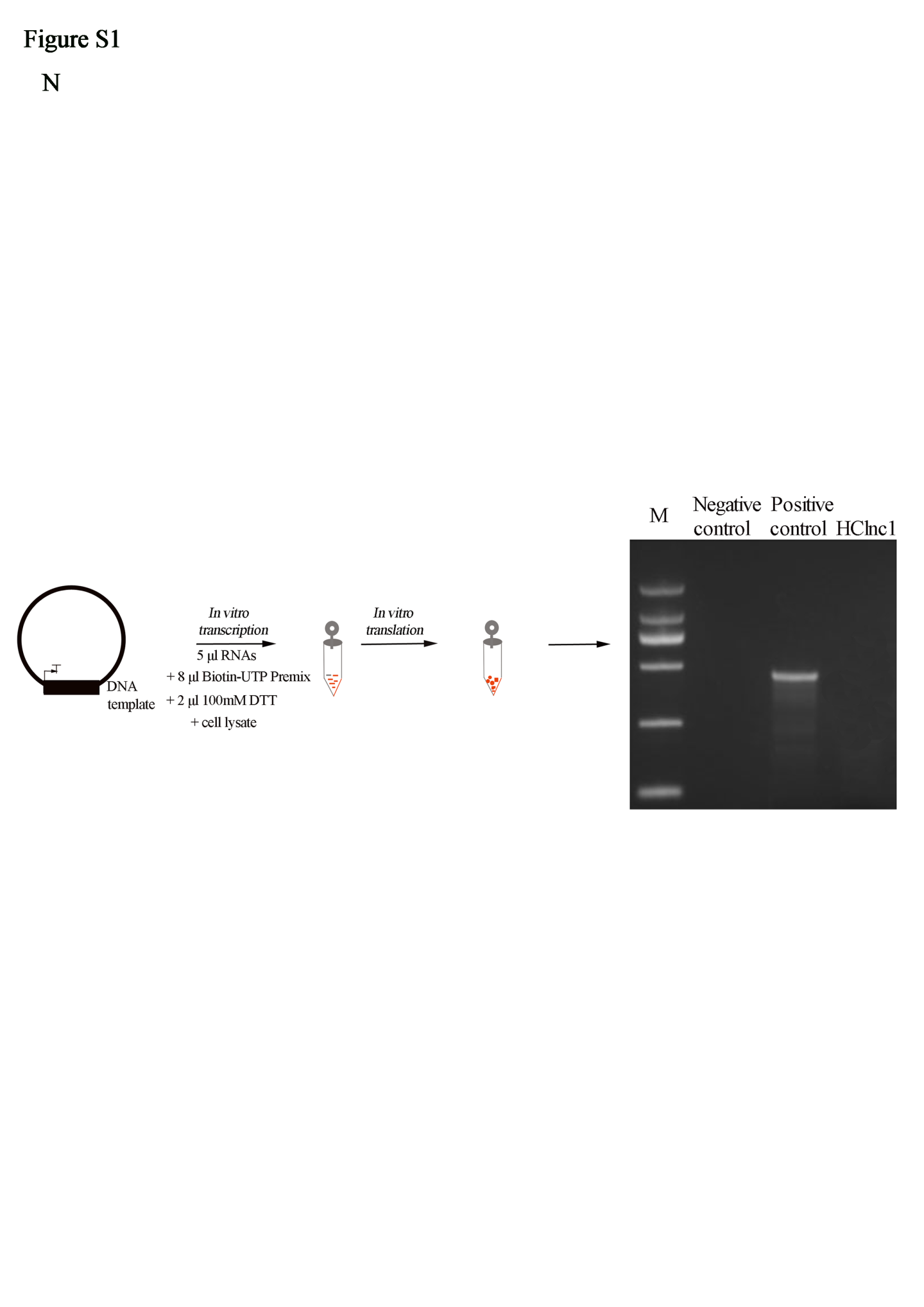


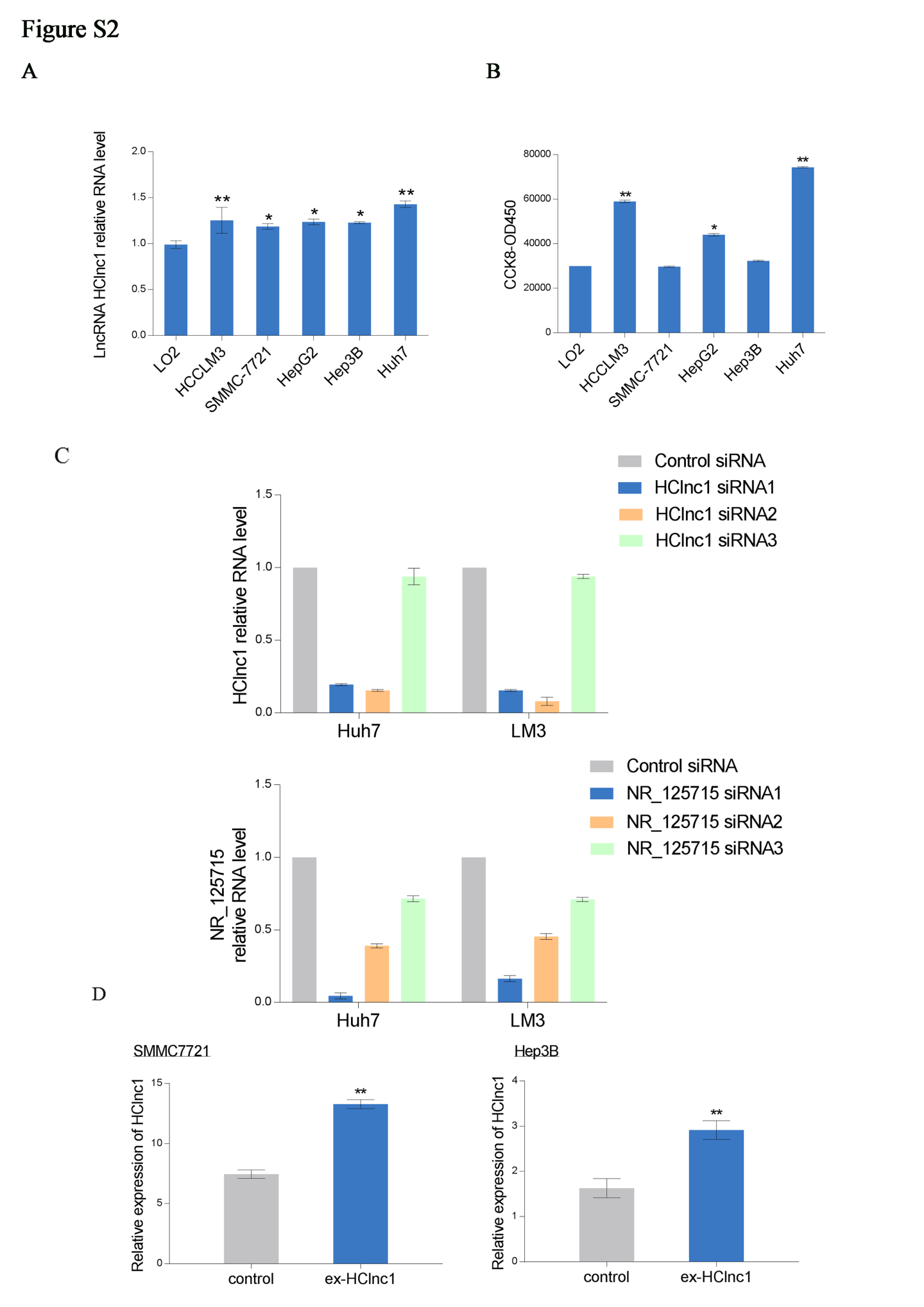


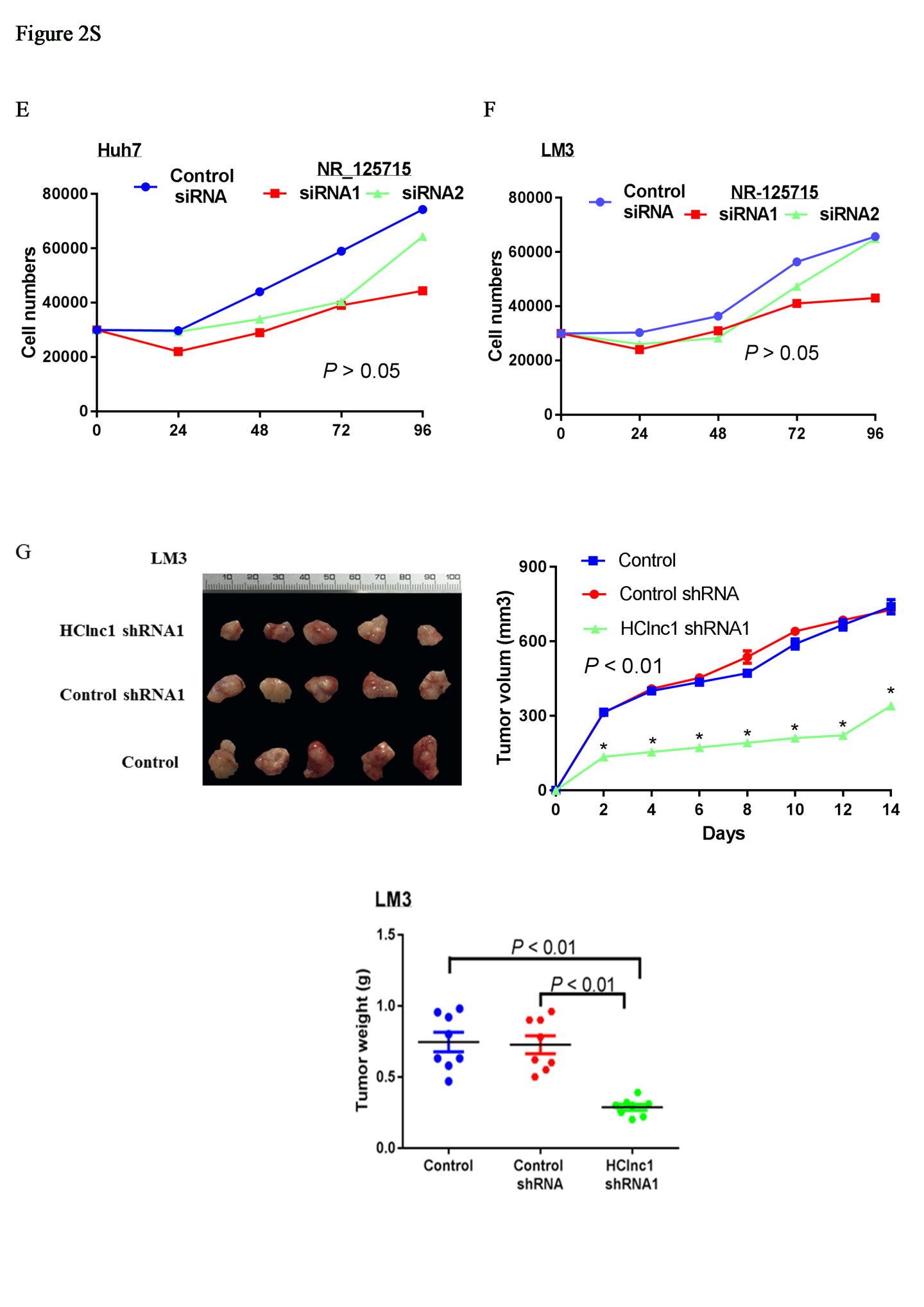


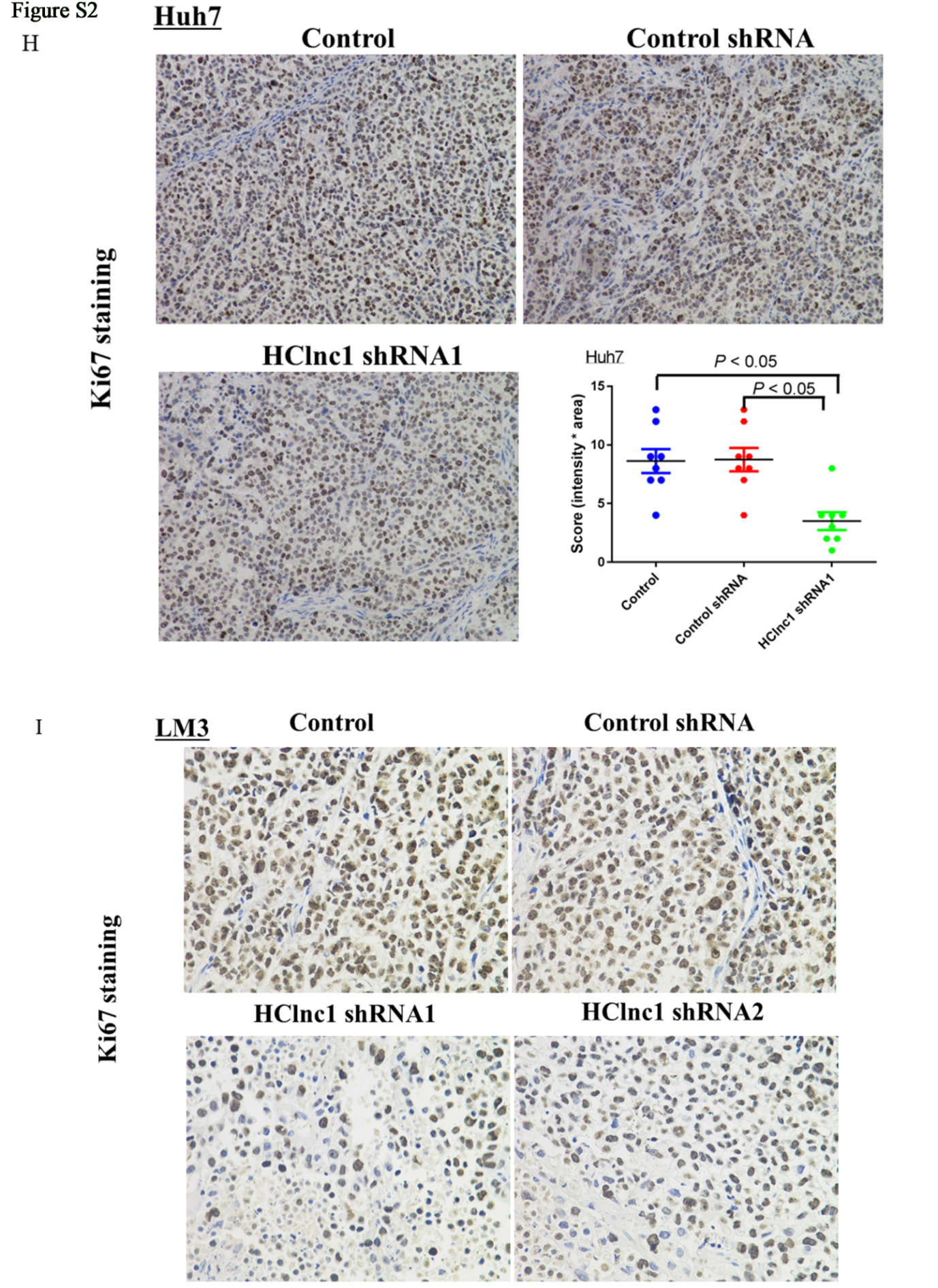


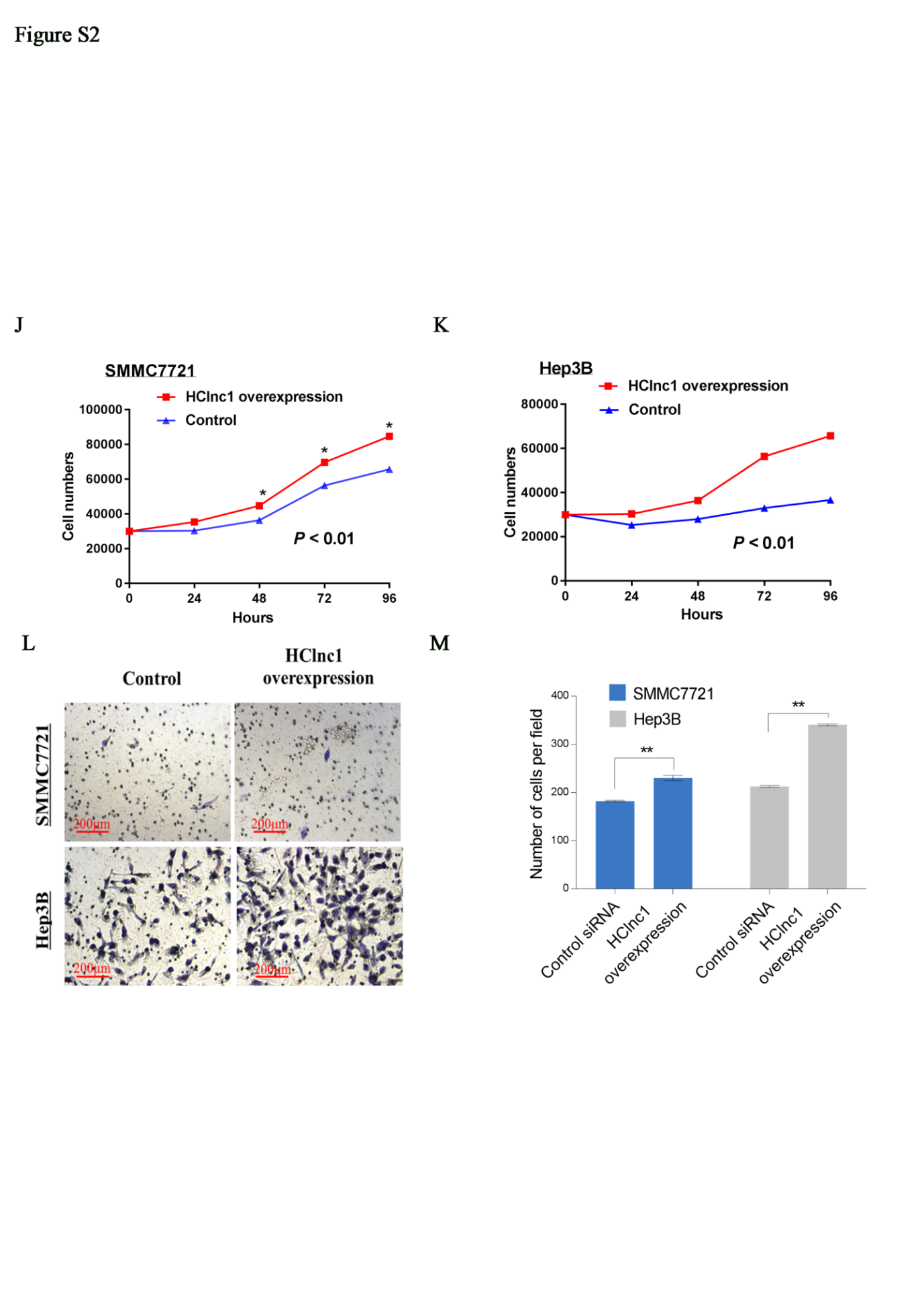


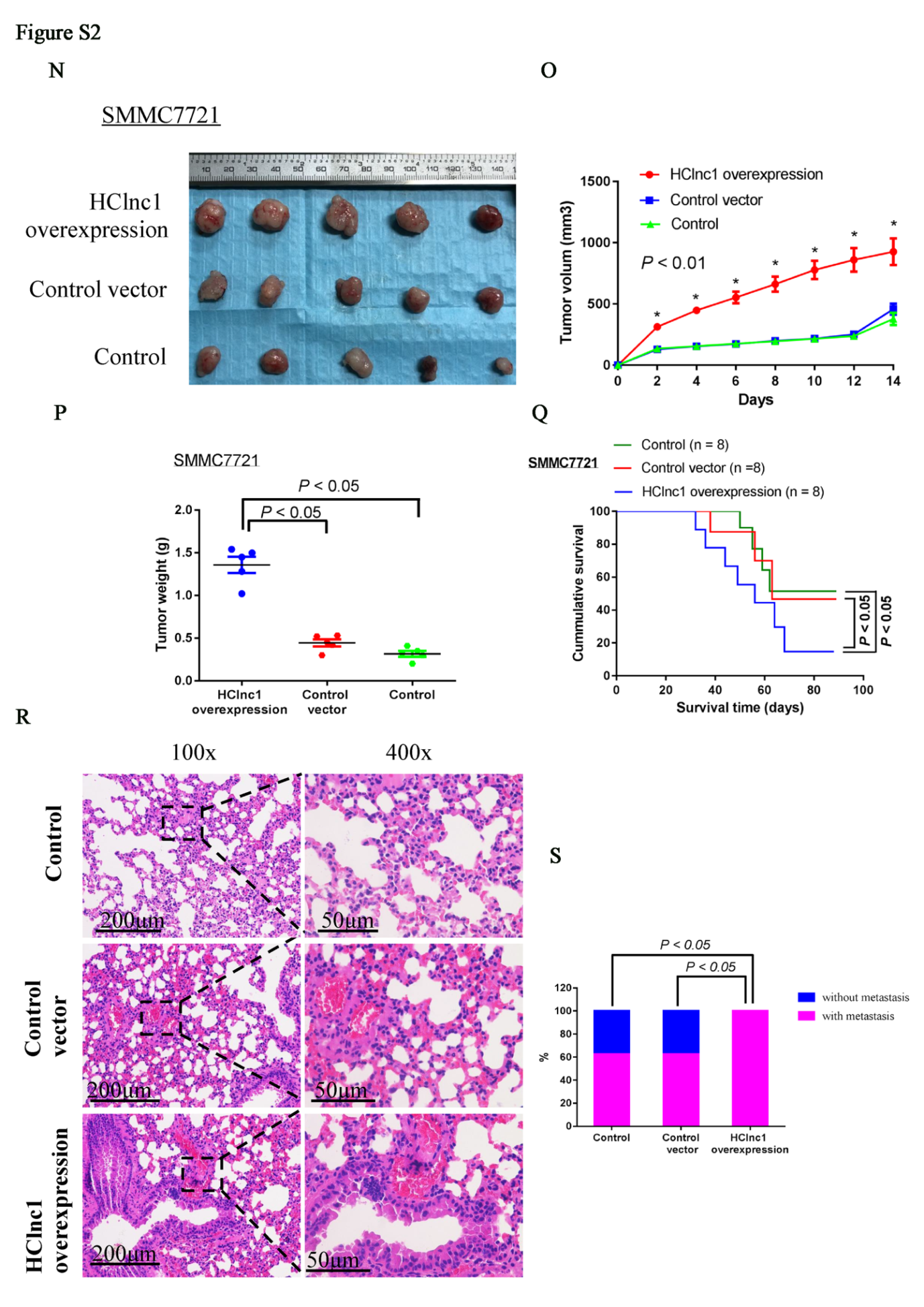


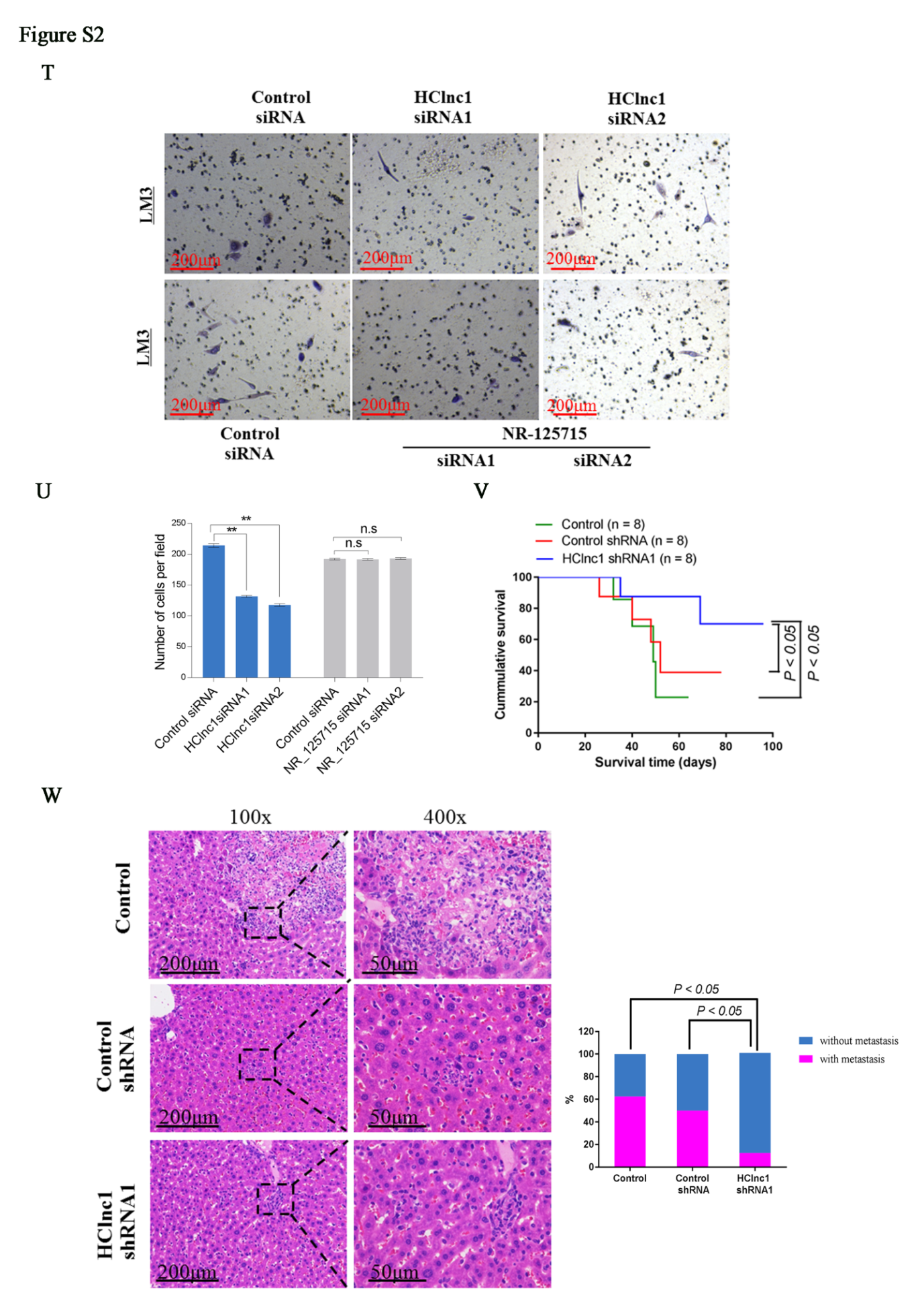


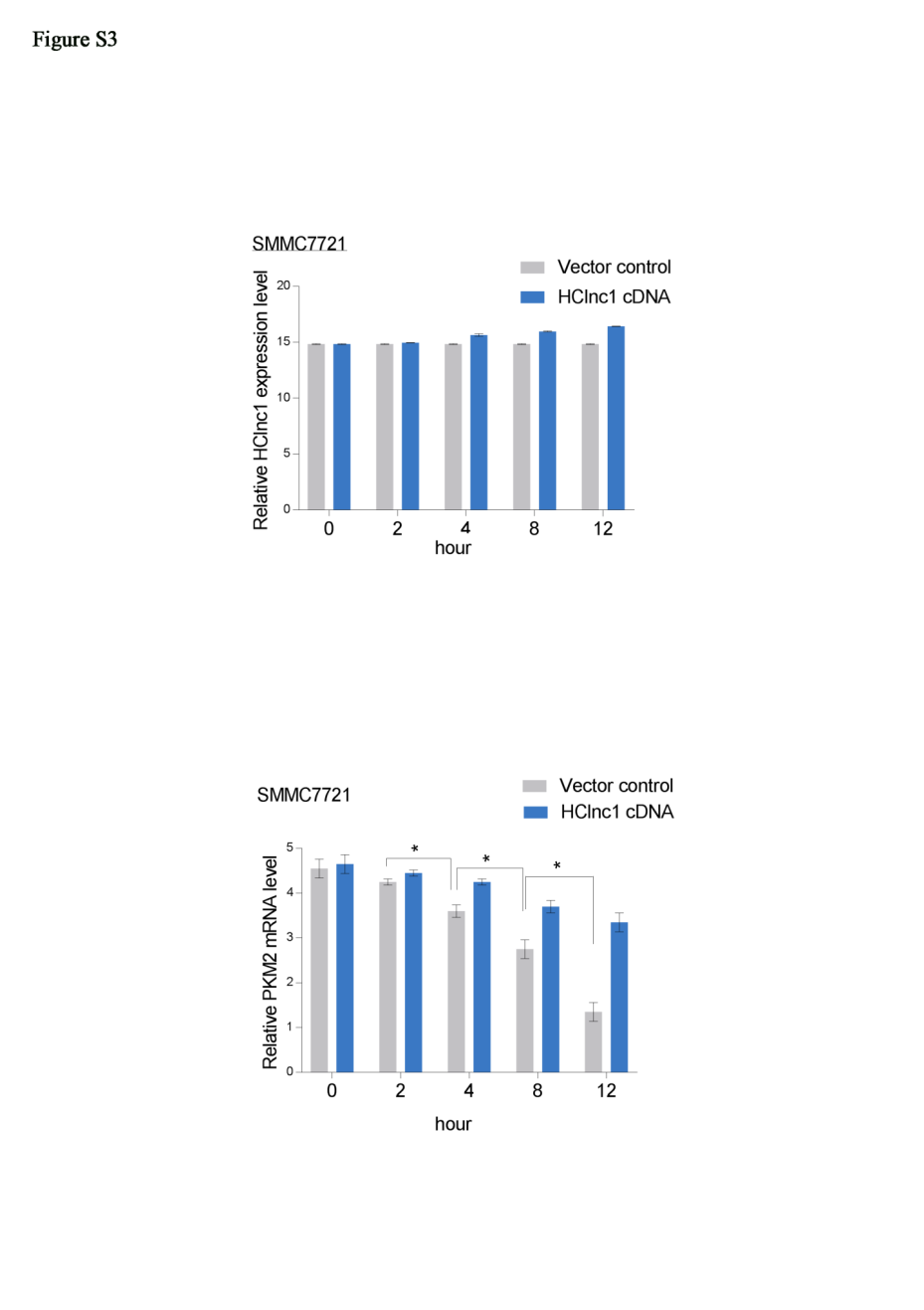


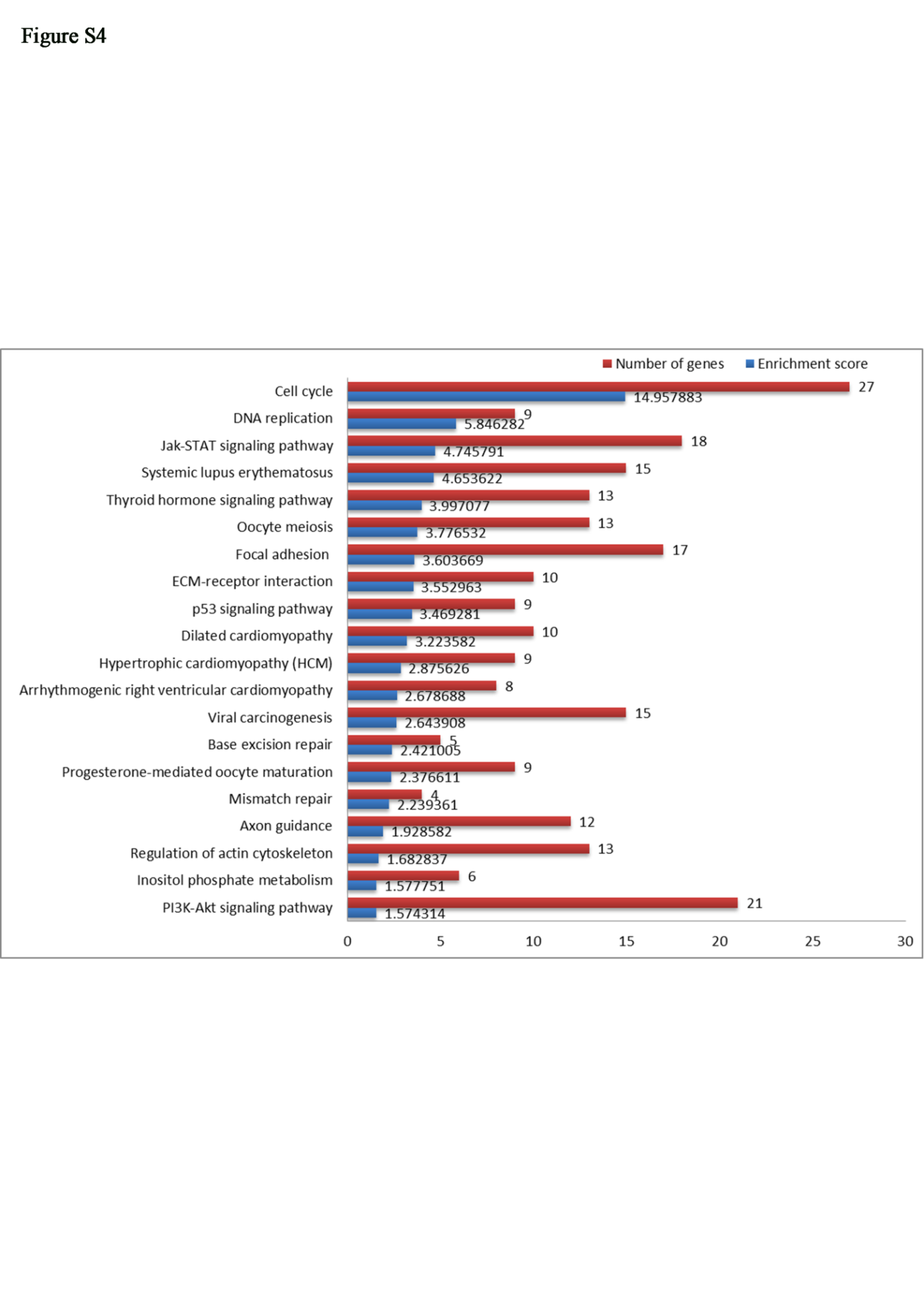


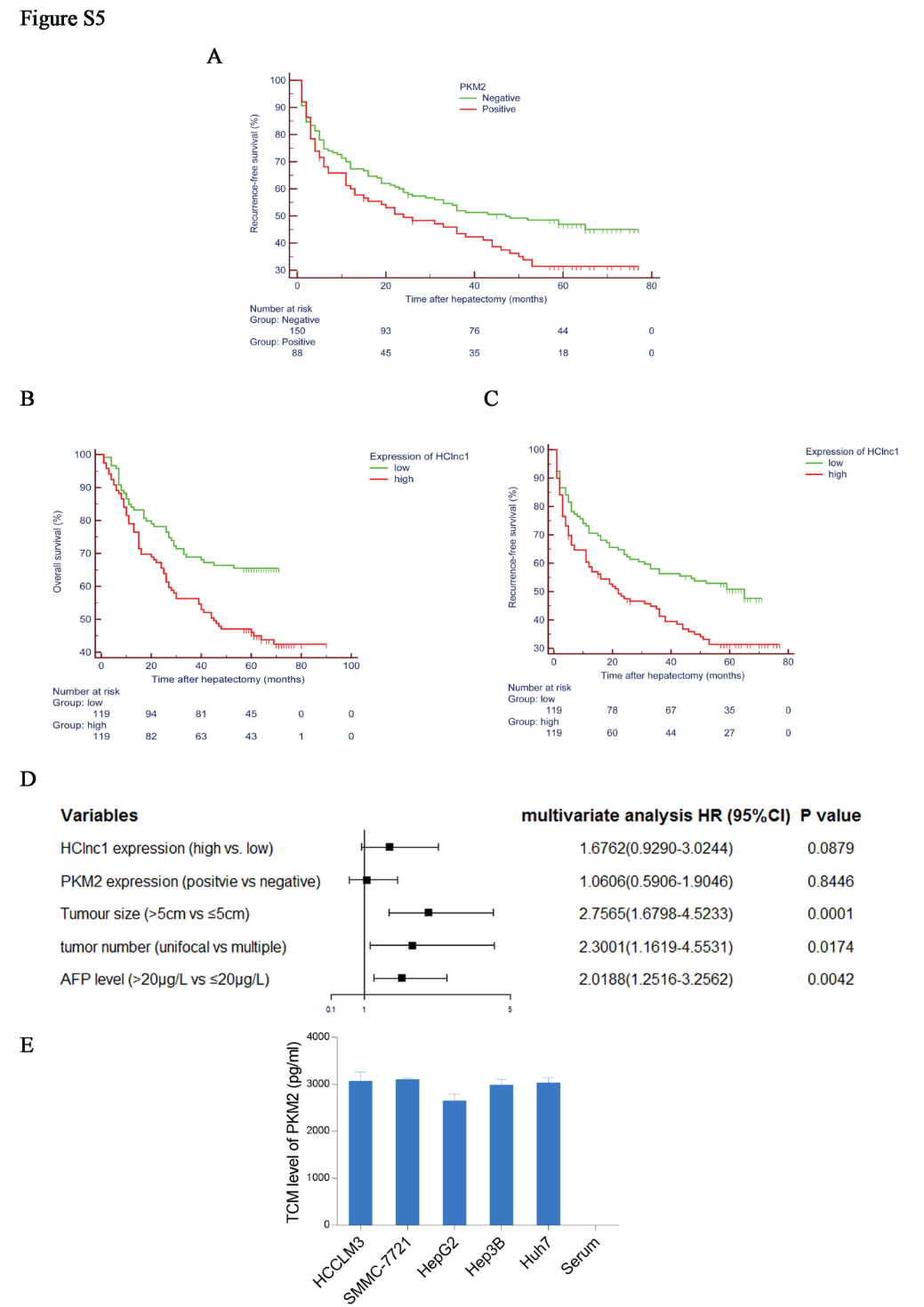


**Supplementary tables**

**Table S1.** Clinical characteristics of 5 HCC patients used for lncRNA array

| Code | Age | Sex | HBsAg | Tumor size (cm) | MVI | Cirrhosis | Pathologic Diagnosis | Serum AFP (µg/L) |
| --- | --- | --- | --- | --- | --- | --- | --- | --- |
| 187694 | 41 | Female | + | 7.2×4.3 | Yes | No | HCC, III | 1210.0 |
| 189554 | 43 | Male | + | 9.1×6.3 | Yes | No | HCC, III | 4.0 |
| 188725 | 73 | Male | + | 6.4×5.2 | No | Micro-nodular | HCC, III | 1210.0 |
| 189618 | 67 | Female | + | 12.7×11.6 | Yes | No | HCC, III-IV | 10.2 |
| 187444 | 61 | Male | + | 6.3×4.2 | No | No | HCC, III | 1167.0 |

Abbreviations: HCC, hepatocellular carcinoma; HBsAg, hepatitis B surface antigen; MVI, microvascular invasion; AFP, α-fetoprotein; C, cancerous tissues; N, noncancerous liver tissues.

Notes: all the patients with negative anti-HCV antibodies in serum.

**Table S2.** Sequences of probes, primers and siRNA used

| **Primers/Probes** | | **Sequence** |
| --- | --- | --- |
| lncRNA-NR_003573-RT-F | | 5’-TAAAGGAGACCTGGAAAATGC-3’ |
| lncRNA-NR_003573-RT-R | | 5’-CTGCGGGAGACCATGATTC-3’ |
| lncRNA-ENST00000558391-RT-F | | 5’-GACTCCTATGTGGGCGATGA-3’ |
| lncRNA-ENST00000558391-RT-R | | 5’-AGTGCAGAGGCTCCGAGAAT-3’ |
| lncRNA-NR_001446-RT-F | | 5’-TCAACAACCAACCAGGAGC-3’ |
| lncRNA-NR_001446-RT-R | | 5’-TCATAATCAACGACAGAGCCAT-3’ |
| lncRNA-NR_002734-RT-F | | 5’-TAAGGAAAACGAAGAACCAGG-3’ |
| lncRNA-NR_002734-RT-R | | 5’-TTTAGGTAAGGATGTGGGAGC-3’ |
| lncRNA-NR_024206-RT-F | | 5’-CCCTCCAGCACCTCTACCT-3’ |
| lncRNA-NR_024206-RT-R | | 5’-ACCAGCCCATGACCAAAAT-3’ |
| lncRNA-NR_027001-RT-F | | 5’-GAGGGTCACGGGTCAACAA-3’ |
| lncRNA-NR_027001-RT-R | | 5’-GTCTAAACTAATGGGGTAGTGGTAG-3’ |
| lncRNA-uc021pbg.1-RT-F | | 5’-CTACCTGAGCCAGTTCTCCTAAA-3’ |
| lncRNA-uc021pbg.1-RT-R | | 5’-CGGGTTCCTCATCGGTGTA-3’ |
| lncRNA-NR_125715-RT-F | | 5’-AGGTATCCTCATGCTGGGGT-3’ |
| lncRNA-NR_125715-RT-R | | 5’-CTTTGGATTCCCGCCTCCTT-3’ |
| lncRNA-ENST00000603052-RT-F | | 5’-CCAGATGAGAAAGAGCTGCCA-3’ |
| lncRNA-ENST00000603052-RT-R | | 5’-AGTGCAGAGGCTCCGAGAAT-3’ |
| lncRNA-NR_024205-RT-F | | 5’-CCCTCCAGCACCTCTACCT-3’ |
| lncRNA-NR_024205-RT-R | | 5’-CAGACAAATGGGAAACCGAC-3’ |
| Human-β-actin-F^▲^ | | 5’-TCATCACCATTGGCAATGAG-3’ |
| Human-β-actin-R^▲^ | | 5’-CACTGTGTTGGCGTACAGGT-3’ |
| MCL1-F | | 5’-CATTCCTGATGCCACCTTCT-3’ |
| MCL1-R | | 5’-TCGTAAGGACAAAACGGG-3’ |
| BIRC5-F | | 5’-CTTTCTCCGCAGTTTCCTCA-3’ |
| BIRC5-R | | 5’-TTGGTGAATTTTTGAAACT-3’ |
| CCND1-F | | 5’-GGCGGATTGGAAATGAACT-3’ |
| CCND1-R | | 5’-TCCTCTCCAAAATGCCAGA-3’ |
| BCL2L1-F | | 5’-GGTAGGAGCTGTGGCGACT-3’ |
| BCL2L1-R | | 5’-CAGGCCTCCTGTGGGAC-3’ |
| CDH2-F | | 5’-TTTTGCCCCCAATCCTAAGA-3’ |
| CDH2-R | | 5’-CAGCGTTCCTGTTCCACTC-3’ |
| MMP2-F | | 5’-TGGATGATGCCTTTGCTCGT-3’ |
| MMP2-R | | 5’-ATCGTCATCAAAATGGGAG-3’ |
| MMP9-F | | 5’-ACGCCCATTTCGACGATGA-3’ |
| MMP9-R | | 5’-GTAGTTGGCCGTGGTACTG-3’ |
| **siRNA sequence** | | **Sequence** |
| NR_125715-homo-204 | Sense | 5’- GCAAGGAUUUAGGGUUCUATT-3’ |
|  | Antisense | 5’- UAGAACCCUAAAUCCUUGCTT-3’ |
| NR_125715-homo-564 | Sense | 5’- GCUAAAGUUUACCUUGCUUTT-3’ |
|  | Antisense | 5’- AAGCAAGGUAAACUUUAGCTT-3’ |
| NR_125715-homo-742 | Sense | 5’- GGCGGGAAUCCAAAGUGAATT-3’ |
|  | Antisense | 5’- UUCACUUUGGAUUCCCGCCTT-3’ |
| NR_125715-homo-875 | Sense | 5’-GCCCUUGUGUUGGAUGUAATT-3’ |
|  | Antisense | 5’-UUACAUCCAACACAAGGGCTT-3’ |
| ENST603052-homo-142 | Sense | 5’-CCUGUCAAAUGCAGGCCAUTT-3’ |
|  | Antisense | 5’-AUGGCCUGCAUUUGACAGGTT-3’ |
| ENST603052-homo-252 | Sense | 5’- GCAACCAGGAGAAACAGAATT-3’ |
|  | Antisense | 5’- UUCUGUUUCUCCUGGUUGCTT-3’ |
| ENST603052-homo-36 | Sense | 5’- GCUCUGUGUCACUAUGUACTT-3’ |
|  | Antisense | 5’- GUACAUAGUGACACAGAGCTT-3’ |
| GAPDH Positive control | Sense | 5’-UGACCUCAACUACAUGGUUTT-3’ |
|  | Antisense | 5’-AACCAUGUAGUUGAGGUCATT-3’ |
| Negative control | Sense | 5’-UUCUCCGAACGUGUCACGUTT-3’ |
|  | Antisense | 5’-ACGUGACACGUUCGGAGAATT-3’ |
| 5’ RACE | P1 | 5’-CAGCCCTGATGGAGAGTGCAGAGG-3’ |
|  | P2 | 5’-CTCTTTCTCATCTGGGCCATGTCC-3’ |
| 3’RACE | P1 | 5’-CCTGTCAAATGCAGGCCATGCAGA-3’ |
|  | P2 | 5’-GCCGAGAGTTCCAGATAACAGAGT-3’ |
| Si-PKM2-1 | Sense | 5’-GGAUGUUGAUAUGGUGUU-3’ |
|  | Antisense | 5’-AAACACCAUAUCAACAUC-3’ |
| Si-PKM2-2 | Sense | 5’-GGCUGUGGCUCUAGACAC-3’ |
|  | Antisense | 5’-UUUAGUGUCUAGAGCCAC-3’ |
| Si-PKM2-3 | Sense | 5’-GCUGUGGCUCUAGACACU-3’ |
|  | Antisense | 5’-UAGUGUCUAGAGCCACAG-3’ |

^▲^ β-actin represents primer for internal control when detected expression of related genes using SYBY-Green method.

**Sequences of primers for shRNA of HClnc1 (ENST00000603052)**

|  | **Sequences** |
| --- | --- |
| B4302-1 | AGGGTTCCAAGCTTAAGCGGCCGCCTTA |
|  | CTTATATATCAAAGTTTCCCTCATAATGTC |
| B4302-2 | TGGAGGACAGGGGAGGGTACATAGTGACAC |
|  | AGAGCAGACATTATGAGGGAAACTTTGATATATAA |
| B4302-3 | CCCTCCCCTGTCCTCCATTCCCCACACCAAGA |
|  | GTATTATGAGTATTAAATGCCACGTGGGCAGGT |
| B4302-4 | CATTCTGCATGGCCTGCATTTGACAGGCCCACC |
|  | CCCTCATTGCCTGGGGAACCTGCCCACGTGGC |
| B4302-5 | TGCAGGCCATGCAGAATGCCGCAGGGCGGGAG |
|  | ACGGGTGAGCCAAGGGAGCCGAGAGTTCCAGAT |
| B4302-6 | TCCTGGTTGCTGGACGCGGGCCATGGCATTTGC |
|  | TGTGCACTCTGTTATCTGGAACTCTCGGCTCC |
| B4302-7 | GCGTCCAGCAACCAGGAGAAACAGAACTGCT |
|  | GAGCTCAGAATAGGCCAAGGCCGCGTCTGCTAGC |
| B4302-8 | GAGGGATGGGGCAGAAGAGGCCAGACAGCA |
|  | GGCTGTGCCCTTGCTCCCTGCTAGCAGACGCGGCC |
| B4302-9 | TCTTCTGCCCCATCCCTCCACCGGGACATGG |
|  | CCCAGATGAGAAAGAGCTGCCAGACGGCAAGTCA |
| B4302-10 | CCGAGAATCCACTCTTGCATAAAACGTTGTC |
|  | CTCTCCCGCCCCACGCTTGACTTGCCGTCTGGCA |
| B4302-11 | TATGCAAGAGTGGATTCTCGGAGCCTCTGCA |
|  | CTCTCCATCAGGGCTGCTGGCATGGAAAACACAG |
| B4302-12 | TTCTGGGAAGTCCAGTAGTTCAATCAAGTCC |
|  | TAGGTGATGGTTTCTCTGTGTTTTCCATGCCAGC |
| B4302-13 | GAACTACTGGACTTCCCAGAAGAGTCCAATT |
|  | TAAAGCAAAGAGGTCCAGAGAAAAAAGCATTCTA |
| B4302-14 | ATCAGTAGAGAGTGTCGGATCCAAATATATA |
|  | AAAATGTCACTAGAATGCTTTTTTCTCTGGACC |

**Table S3.** Clinicopathological characteristic of HCC patients included

| Variable | Cohort1 | Cohort2 | Cohort3 | Cohort4 | *p* value |
| --- | --- | --- | --- | --- | --- |
| All | 60 | 80 | 120 | 238 |  |
| Age | 54.8 ± 10.9 | 51.1 ± 10.1 | 50.7 ± 11.3 | 51.4 ± 10.9 | 0.10 |
| Age>50, n (%) | 36 (60.0%) | 40 (50.0%) | 63 (52.5%) | 126 (52.9%) | 0.69 |
| Gender: male, n (%) | 50 (83.3%) | 68 (85.0%) | 106 (88.3%) | 211 (89.8%) | 0.43 |
| HBs antigen: +, n (%) | 54 (90.0%) | 59 (73.8%) | 102 (85.0%) | 201 (84.5%) | 0.05 |
| Cirrhosis: +, n (%) | 36 (60.0%) | 51 (63.8%) | 71 (59.2%) | 158 (66.4%) | 0.54 |
| AFP(ug/L) >20, n (%) | 37 (61.7%) | 51 (63.8%) | 87 (72.5%) | 160 (67.2%) | 0.42 |
| Tumor size (cm) | 7.3 ± 4.0 | 7.3 ± 3.9 | 6.9 ± 4.6 | 6.7 ± 3.9 | 0.63 |
| Tumor size (cm)>5, n (%) | 42 (70.0%) | 47 (58.8%) | 66 (55.0%) | 130 (54.6%) | 0.17 |
| No. of tumor: multiple, n (%) | 12 (20.0%) | 18 (22.5%) | 24 (20.0%) | 54 (22.7%) | 0.92 |
| Edmondson grade: III+ IV, n (%) | 55 (91.7%) | 73 (91.3%) | 110 (91.7%) | 219 (92.0%) | 0.99 |
| Microvascular invasion: +, n (%) | 36 (60.0%) | 54 (67.5%) | 69 (57.5%) | 152 (63.9%) | 0.48 |
| Child pugh A, n (%) | 54 (90.0%) | 74 (92.5%) | 105 (87.5%) | 216 (90.8%) | 0.67 |
| BCLC stage |  |  |  |  | 0.89 |
| A+B | 54 (90.0%) | 71 (88.8%) | 106 (88.3%) | 216 (90.8%) |  |
| C | 6 (10.0%) | 9 (11.3%) | 14 (11.7%) | 22 (9.2%) |  |
| TNM stage |  |  |  |  | 0.70 |
| I | 20 (33.3%) | 25 (31.3%) | 37 (30.8%) | 73 (30.7%) |  |
| II | 25 (41.7%) | 34 (42.5%) | 44 (36.7%) | 108 (45.4%) |  |
| III+IV | 15 (25.0%) | 21 (26.3%) | 39 (32.5%) | 57 (24.0%) |  |

**Table S4.** Univariate analysis of overall survival of 120 patients with HCC

| **Variable** | **OS^▲^ (no-event)** | **OS^▲^ (event)** | **P value** |
| --- | --- | --- | --- |
| Age | 50.7(10.3) | 50.6(12.0) | 0.595 |
| Age: >55/<55 | 0.35(0.48) | 0.35(0.48) | 0.954 |
| Gender: male/female | 39/7 | 67/7 | 0.383 |
| HBs antigen: +/- | 0.78(0.42) | 0.89(0.31) | 0.193 |
| Liver cirrhosis: +/- | 27/19 | 44/30 | 0.956 |
| Serum albumin (g/L) | 41.8(4.18) | 40.6(4.56) | 0.143 |
| Serum albumin (g/L): >40/≤40 | 0.57(0.50) | 0.53(0.50) | 0.679 |
| Serum bilirubin (μmol/L) | 16.7(7.75) | 16.8(6.72) | 0.479 |
| Serum bilirubin (μmol/L): >17/≤17 | 0.24(0.43) | 0.28(0.45) | 0.259 |
| HBe antigen: +/- | 0.24(0.43) | 0.26(0.44) | 0.473 |
| Tumor size (cm) | 5.31(3.83) | 7.90(4.82) | <0.001^★^ |
| Tumor size (cm): >5/≤5 | 0.37(0.49) | 0.66(0.48) | <0.001^★^ |
| Number of tumor: Solitary/Multiple | 41/5 | 55/19 | 0.003^★^ |
| Edmondson Grade |  |  | 0.930 |
| I | 3 | 4 |  |
| II | 1 | 2 |  |
| III | 42 | 66 |  |
| IV | 0 | 2 |  |
| Micro-vascular invasion: +/- | 27/19 | 42/32 | 0.860 |
| Macro-vascular invasion: +/- | 0.04(0.21) | 0.16(0.37) | <0.001^★^ |
| Satellite: +/- | 7/39 | 10/64 | 0.844 |
| Encapsulation: complete/- | 29/17 | 43/31 | 0.419 |
| TNM stage |  |  | 0.012^★^ |
| I | 24 | 18 |  |
| II | 13 | 26 |  |
| III | 9 | 28 |  |
| Ⅳ | 0 | 2 |  |
| BCLC stage |  |  | <0.001^★^ |
| A | 39 | 49 |  |
| B | 5 | 13 |  |
| C | 2 | 12 |  |
| ALT (U/L) | 61.9(44.9) | 69.9(64.0) | 0.447 |
| ALT (U/L): >40/≤40 | 0.57(0.50) | 0.62(0.49) | 0.518 |
| AFP (μg/L) | 407(459) | 1394(7293) | 0.394 |
| AFP (μg/L): >20/ ≤20 | 0.63(0.49) | 0.78(0.41) | 0.035^★^ |
| LncRNA-HClnc1 | 0.46(0.50) | 0.61(0.49) | 0.038^★^ |

▲ The time follow-up ended is used to calculate the Overall Survival

^★^P<0.05 by Long-rank test.

OS: Overall Survival; ^*^the time follow-up ended is used to calculate the Recurrence-Free Survival and Overall survival; p value by Long-rank test less than 0.05 was considered statistical significant.

**Table S5.** Univariate analysis of recurrence-free survival of 120 patients with HCC

| **Variable** | **RFS^▲^ (no-event)** | **RFS^▲^ (event)** | **P value** |
| --- | --- | --- | --- |
| Age | 51.4(11.1) | 50.3(11.4) | 0.214 |
| Age: >55/<55 | 0.39(0.49) | 0.33(0.47) | 0.465 |
| Gender: male/female | 29/7 | 77/7 | 0.140 |
| HBs antigen: +/- | 0.75(0.44) | 0.89(0.31) | 0.095 |
| Liver cirrhosis: +/- | 20/16 | 51/33 | 0.591 |
| Serum albumin (g/L) | 41.8(3.94) | 40.8(4.62) | 0.187 |
| Serum albumin (g/L): >40/≤40 | 0.56(0.50) | 0.54(0.50) | 0.655 |
| Serum bilirubin (μmol/L) | 15.7(7.13) | 17.2(7.08) | 0.229 |
| Serum bilirubin (μmol/L): >17/≤17 | 0.19(0.40) | 0.30(0.46) | 0.141 |
| HBe antigen: +/- | 0.25(0.44) | 0.25(0.44) | 0.605 |
| Tumor size (cm) | 4.84(3.00) | 7.79(4.92) | <0.001 |
| Tumor size (cm): >5/≤5 | 0.31(0.47) | 0.65(0.48) | <0.001 |
| Number of tumor: Solitary/Multiple | 32/4 | 64/20 | 0.008 |
| Edmondson Grade |  |  | 0.907 |
| I | 1 | 6 |  |
| II | 1 | 2 |  |
| III | 34 | 74 |  |
| Ⅳ | 0 | 2 |  |
| Micro-vascular invasion: +/- | 20/16 | 49/35 | 0.744 |
| Macro-vascular invasion: +/- | 0.03(0.17) | 0.15(0.36) | 0.002 |
| Satellite: +/- | 7/29 | 10/74 | 0.380 |
| Encapsulation: complete/- | 24/12 | 48/36 | 0.173 |
| TNM stage |  |  | 0.018 |
| I | 20 | 22 |  |
| II | 9 | 30 |  |
| III | 7 | 30 |  |
| IV | 0 | 2 |  |
| BCLC stage |  |  | 0.001 |
| A | 31 | 57 |  |
| B | 4 | 14 |  |
| C | 1 | 13 |  |
| ALT (U/L) | 67.9(48.1) | 66.4(61.1) | 0.854 |
| ALT (U/L): >40/≤40 | 0.64(0.49) | 0.58(0.50) | 0.979 |
| AFP (μg/L) | 446(471) | 1260(6850) | 0.669 |
| AFP (μg/L): >20/ ≤20 | 0.67(0.48) | 0.75(0.44) | 0.083 |
| LncRNA-HClnc1 | 0.47(0.51) | 0.58(0.50) | 0.046 |

▲The time follow-up ended is used to calculate the Recurrence-free Survival

^★^P<0.05 by Long-rank test.

RFS: Recurence-free Survival; ^*^the time follow-up ended is used to calculate the Recurrence-Free Survival and Overall survival; p value by Long-rank test less than 0.05 was considered statistical significant.

**Table S6.** Multivariate analysis for factors related to HCC overall survival

| variable | Univariate analysis | | | Multivariate analysis | | |
| --- | --- | --- | --- | --- | --- | --- |
|  | Hazard ratio | CI (95%) | p value | Hazard ratio | CI (95%) | p value |
| Age(year) | 0.99 | 0.97-1.02 | 0.595 |  |  |  |
| Age(year) >55/ ≤55 | 0.99 | 0.61-1.59 | 0.954 |  |  |  |
| Gender: male/female | 1.41 | 0.65-3.07 | 0.383 |  |  |  |
| HBsAg: positive/negative | 1.63 | 0.78-3.40 | 0.193 |  |  |  |
| HBeAg: positive/negative | 1.21 | 0.72-2.04 | 0.473 |  |  |  |
| Liver cirrhosis: +/- | 1.01 | 0.64-1.61 | 0.956 |  |  |  |
| TBL (μmol/L) | 1.01 | 0.98-1.04 | 0.479 |  |  |  |
| TBL (μmol/L): >17 / ≤17 | 1.34 | 0.81-2.23 | 0.259 |  |  |  |
| ALB (g/dL) | 0.96 | 0.91-1.01 | 0.143 |  |  |  |
| ALB (g/dL):>40 / ≤40 | 0.91 | 0.58-1.43 | 0.679 |  |  |  |
| ALT (U/L) | 1.00 | 1.00-1.01 | 0.447 |  |  |  |
| ALT (U/L): >40 / ≤40 | 1.17 | 0.73-1.87 | 0.518 |  |  |  |
| TNM stage |  |  | 0.001^★^ | 1.87 | 1.13-3.11 | 0.016^★^ |
| I | Ref. | Ref. |  |  |  |  |
| II | 2.06 | 1.16-3.66 | 0.013 |  |  |  |
| III | 2.37 | 1.25-4.48 | 0.008 |  |  |  |
| IV | 10.6 | 2.33-48.5 | 0.002 |  |  |  |
| Number of tumor: Solitary/Multiple | 0.46 | 0.27-0.78 | 0.003^★^ |  |  |  |
| Edmondson grade |  |  | 0.930 |  |  |  |
| I | Ref. | Ref. |  |  |  |  |
| II | 1.17 | 0.21-6.42 | 0.854 |  |  |  |
| III | 1.10 | 0.40-3.03 | 0.847 |  |  |  |
| IV | 1.73 | 0.31-9.60 | 0.533 |  |  |  |
| Encapsulation: complete/- | 0.82 | 0.52-1.31 | 0.419 |  |  |  |
| Micro-vascular invasion:+/- | 1.04 | 0.66-1.65 | 0.860 |  |  |  |
| Macro-vascular invasion:+/- | 3.21 | 1.71-6.04 | <0.001^★^ |  |  |  |
| AFP(ug/L) | 1.00 | 1.00-1.00 | 0.394 |  |  |  |
| AFP: ≤20ug/L vs>20ug/L | 1.81 | 1.04-3.16 | 0.035^★^ |  |  |  |
| Tumor size(cm) | 1.10 | 1.06-1.15 | <0.001^★^ |  |  |  |
| Tumor size: >5cm vs AFP: ≤5cm | 2.74 | 1.68-4.46 | <0.001^★^ |  |  |  |
| Satellite: presence/absence | 0.93 | 0.48-1.82 | 0.844 |  |  |  |
| LncRNA HClnc1 | 1.64 | 1.03-2.62 | 0.038^★^ | 1.78 | 1.11-2.86 | 0.017^★^ |

^★^P<0.05 by Long-rank test. HCC: Hepatocellular carcinoma; HR: Hazard ratio; CI: Confidence interval; TBL: Total bilirubin; ALB: Albumin; ALT: Alanine aminotransferase; PT: Prothrombin time; PLT: Blood platelet; AFP: Alpha-fetoprotein.

**Table SV7.** Multivariate analysis for factors related to HCC recurrence-free survival

| variable | Univariate analysis | | | Multivariate analysis | | |
| --- | --- | --- | --- | --- | --- | --- |
|  | Hazard ratio | CI (95%) | p value | Hazard ratio | CI (95%) | p value |
| Age(year) | 0.99 | 0.97-1.01 | 0.214 |  |  |  |
| Age(year) >55/ ≤55 | 0.84 | 0.54-1.33 | 0.465 |  |  |  |
| Gender: male/female | 1.77 | 0.81-3.84 | 0.149 |  |  |  |
| HBsAg: positive/negative | 1.80 | 0.90-3.60 | 0.095 | 2.13 | 1.05-4.29 | 0.035 |
| HBeAg: positive/negative | 1.14 | 0.70-1.87 | 0.605 |  |  |  |
| Liver cirrhosis: +/- | 1.13 | 0.73-1.74 | 0.591 |  |  |  |
| TBL (μmol/L) | 1.02 | 0.99-1.05 | 0.229 |  |  |  |
| TBL (μmol/L): >17 / ≤17 | 1.42 | 0.89-2.28 | 0.141 |  |  |  |
| ALB (g/dL) | 0.97 | 0.92-1.02 | 0.187 |  |  |  |
| ALB (g/dL):>40 / ≤40 | 0.91 | 0.59-1.39 | 0.655 |  |  |  |
| ALT (U/L) | 1.00 | 1.00-1.00 | 0.854 |  |  |  |
| ALT (U/L): >40 / ≤40 | 1.01 | 0.65-1.55 | 0.979 |  |  |  |
| TNM stage |  |  | 0.001^★^ | 2.09 | 1.30-3.36 | 0.002^★^ |
| I | Ref. | Ref. |  |  |  |  |
| II | 2.02 | 1.18-3.43 | 0.010 |  |  |  |
| III | 2.61 | 1.46-4.66 | 0.001 |  |  |  |
| IV | 17.7 | 3.88-81.2 | <0.001 |  |  |  |
| Number of tumor: Solitary/Multiple | 0.51 | 0.31-0.84 | 0.008^★^ |  |  |  |
| Edmondson grade |  |  | 0.930 |  |  |  |
| I | Ref. | Ref. |  |  |  |  |
| II | 0.66 | 0.13-3.27 | 0.611 |  |  |  |
| III | 0.83 | 0.36-1.90 | 0.655 |  |  |  |
| IV | 1.17 | 0.24-5.81 | 0.848 |  |  |  |
| Encapsulation: complete/- | 0.74 | 0.48-1.15 | 0.173 |  |  |  |
| Micro-vascular invasion:+/- | 1.08 | 0.70-1.66 | 0.744 |  |  |  |
| Macro-vascular invasion:+/- | 2.60 | 1.42-5.74 | 0.002^★^ |  |  |  |
| AFP(ug/L) | 1.00 | 1.00-1.00 | 0.394 |  |  |  |
| AFP: ≤20ug/L vs>20ug/L | 1.55 | 0.94-2.55 | 0.083 |  |  |  |
| Tumor size(cm) | 1.10 | 1.06-1.14 | <0.001^★^ |  |  |  |
| Tumor size: >5cm vs AFP: ≤5cm | 2.85 | 1.81-4.50 | <0.001^★^ |  |  |  |
| Satellite: presence/absence | 0.74 | 0.38-1.44 | 0.380 |  |  |  |
| LncRNA HClnc1 | 1.56 | 1.01-2.41 | 0.046^★^ | 1.73 | 1.11-2.71 | 0.016^★^ |

^★^P<0.05 by Long-rank test. HCC: Hepatocellular carcinoma; HR: Hazard ratio; CI: Confidence interval; TBL: Total bilirubin; ALB: Albumin; ALT: Alanine aminotransferase; PT: Prothrombin time; PLT: Blood platelet; AFP: Alpha-fetoprotein.

**Table S8.** Interactions for overall survival by HClnc1 and TNM stage in patients with HCC

| Variables | Chi-Square | d.f. | *P* value for Interaction▲ |
| --- | --- | --- | --- |
| HClnc1 | 10.17 | 2 | 0.0062 |
| TNM stage | 26.82 | 2 | <0.0001 |
| HClnc1*TNM stage | 4.49 | 1 | 0.0342 |

▲P values for interaction were obtained using a postestimation Wald test to generate an omnibus P value for the interaction term between HClnc1 and TNM stage.

**Table S9.** Interactions for recurrence survival by HClnc1 and TNM stage in patients with HCC

| Variables | Chi-Square | d.f. | *P* value for Interaction▲ |
| --- | --- | --- | --- |
| HClnc1 | 14.83 | 2 | 0.0006 |
| TNM stage | 35.19 | 2 | <0.0001 |
| HClnc1*TNM stage | 8.24 | 1 | 0.0041 |

▲P values for interaction were obtained using a postestimation Wald test to generate an omnibus P value for the interaction term between HClnc1 and TNM stage.

**Table S10.** A list of proteins identified by mass spectrometry.

| **Hits** | **Protein** | **No. peptide** | **Description** |
| --- | --- | --- | --- |
| 1 | Pyruvate kinase M2 (PKM2) | 10 | In addition to its well-established role in aerobic glycolysis, PKM2 is essential for its epigenetic Regulation of gene expression and tumorigenesis |
| 2 | Plakophilins (PKP1) | 5 | Loss secondary to promoter methylation |
| 3 | Transmembrane protein 33 (TMEM33) | 3 | May function as a determinant of the ER stress-response events in cancer cells and modulator of the unfolded protein response signaling |
| 4 | Serpin peptidase inhibitor, clade (SERPINB12) | 5 | Novel member of the human ov-serpin family that is widely expressed and inhibits trypsin-like serine proteinases |
| 5 | Asp-Glu-Ala-Asp box polypeptide 39 (DDX39 A & B) | 5 | Regulate androgen receptor splice variant AR-V7 generation |

Hits: Numbering of all proteins identified in each sample, listed according to relative abundances.

No. of Peptide: The number of peptides sequenced by LC-MS/MS in each identified protein.
